# Discovery of Novel Imidazothiazole-Based Hydroxamic Acid Derivatives as Potent Indoleamine 2,3-Dioxygenase 1 and Histone Deacetylase 6 Dual Inhibitors

**DOI:** 10.3390/molecules30122508

**Published:** 2025-06-07

**Authors:** Shi Zhang, Yan-Fei Wang, Hai-Rui Lu, Xue-Qin Yang, Ye Zhang, Xian-Li Ma, Ri-Zhen Huang

**Affiliations:** 1 Guangxi Key Laboratory of Drug Discovery and Optimization, School of Pharmacy, Guilin Medical University, Guilin 541199, China; 2 Guangxi Engineering Research Center for Pharmaceutical Molecular Screening and Druggability Evaluation, School of Pharmacy, Guilin Medical University, Guilin 541199, China; 3 Key Laboratory of Medical Biotechnology and Translational Medicine, School of Pharmacy, Guilin Medical University, Guilin 541199, China

**Keywords:** indoleamine 2,3-dioxygenase 1, histone deacetylase 6, dual inhibitors, immunotherapy, hydroxamic acids, imidazo[2,1-b]thiazole

## Abstract

In order to take advantage of both immunotherapeutic and epigenetic antitumor agents, a series of imidazothiazole-based hydroxamic acid derivatives were designed based on the pharmacophore fusion strategy and evaluated as potent IDO1 and HDAC6 dual inhibitors. Among these inhibitors, the most potent compound *3-(4-Bromophenyl)-N-{4-[(7-(hydroxyamino)-7-oxoheptyl)amino]phenyl}imidazo[2,1-b]thiazole-5-carboxamide* (**10e**) showed considerable IDO1 inhibitory activity and a good selectivity profile for HDAC6 over the other HDAC isoforms. The intracellular inhibition of HDAC6 by **10e** was validated by Western blot analysis. Docking studies illustrated that the possible binding modes of compound **10e** interacted with IDO1 and HDAC6. Moreover, compound **10e** was found to arrest the cell cycle at the G2/M phase in HCT-116 cells. In particular, compound **10e** also exhibited potent in vivo antitumor efficacy in CT26 tumor-bearing BALB/c mice models, with no significant toxicity. Collectively, this work provides a promising lead compound that serves as IDO1/HDAC6 dual inhibitor for the development of novel antitumor agents.

## 1. Introduction

Immune checkpoint therapy has emerged as a groundbreaking advancement in cancer immunotherapy since the FDA approval of the CTLA-4 inhibitor ipilimumab and PD-1 blockers pembrolizumab and nivolumab [1]. Despite its success, the clinical benefits of anti-PD-1 monotherapy remain constrained by low response rates, immune-related adverse effects, and additional immunosuppressive factors in the tumor microenvironment (TME) [2,3]. To address these limitations, researchers are increasingly exploring combination approaches, such as integrating immunotherapy with chemotherapy or targeted therapy, some of which have shown promising clinical outcomes [4,5]. In addition, multitargeted agents—capable of modulating multiple pathways to enhance therapeutic efficacy—have garnered significant interest from both the industry and academia [6].

**Figure 1:**
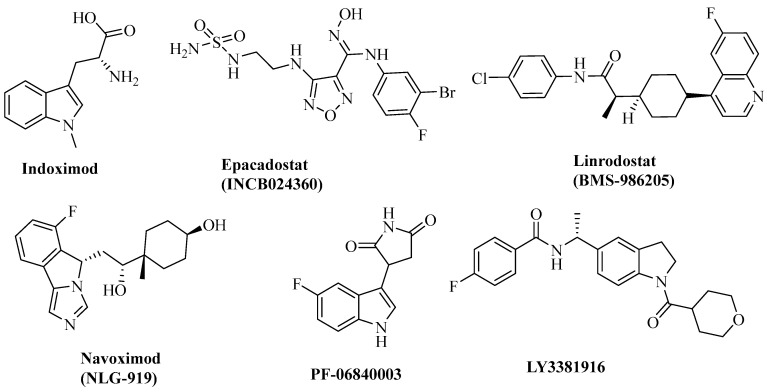
Structures of candidates targeting IDO1 in clinical trial.

Indoleamine 2,3-dioxygenase 1 (IDO1), a heme-containing oxidoreductase that catalyzes tryptophan catabolism to kynurenine in the first rate-limiting step of the kynurenine pathway, plays an important role in tumor evasion of immune surveillance [7,8]. IDO1-induced immune tolerance is widely considered as one of the most critical mechanisms evolved by tumors to escape immune surveillance. It is constitutively overexpressed in many human tumors and host antigen-presenting cells, and has been correlated with different tumor progression parameters and poor prognosis [9]. Furthermore, IDO1 has been found to be involved in the suppression of CD^8+^ T effector cells and natural killer (NK) cells, increasing the activity of CD^4+^ regulatory T cells (Treg) and myeloid-derived suppressor cells (MDSCs) [10,11]. Hence, IDO1 has been considered as an important target for immunotherapeutic intervention. At present, several small-molecule IDO1 inhibitors have entered clinical trials [12,13], such as Navoximod (NLG919), epacadostat, Linrodostat (BMS-986205), PF-06840003, and LY338191616 (Figure 1). In addition, imidazothiazole derivatives have been identified as potent IDO1 inhibitors [14,15]. However, preclinical and clinical studies indicated that IDO1 inhibitors only exhibited moderate antitumor activity when used as single agents [16].

**Figure 2:**
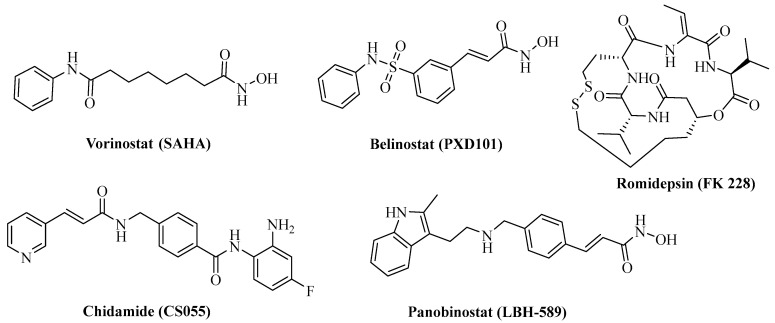
Structures and pharmacophores of representative HDAC inhibitors.

Histone deacetylases (HDACs) are a family of important epigenetic enzymes that are responsible for controlling gene expression by modulating the acetylation status of histone and some nonhistone proteins and play a critical role in various cellular processes, such as transcription, the cell cycle, and cellular metabolism [17,18,19]. To date, five HDAC inhibitors (Figure 2)—Vorinostat, Romidepsin (FK 228), Panobinostat (LBH-589), Belinostat (PXD101), and Chidamide (CS055)—have been approved for the clinical treatment of hematological malignancies [20,21,22]. However, these known HDAC inhibitors are mainly pan-HDAC inhibitors, leading to undesirable side effects such as cardiotoxicity, and show little efficacy in the treatment of solid tumors as single agents [23,24]. HDAC6, predominantly a cytosolic member of the class IIb HDACs, is unique due to its ability to deacetylate a diverse set of nonhistone substrates such as α-tubulin and HSP90 [25]. HDAC6 is highly expressed in various cancer types, including malignant melanoma, bladder cancer, and lung cancer, and thus highlighting it as a potential therapeutic target for cancer treatment. Recent research studies have revealed that the selective inhibition of HDAC6 leads to the inhibition of tumorigenesis and metastasis, and to the increased sensitivity of tumors to other anticancer agents [26,27,28]. Furthermore, HDAC6 inhibitors have been reported to possess immunotherapeutic activity by decreasing programmed death-ligand 1 (PD-L1) expression by deactivating the STAT3 pathway [29,30]. Importantly, recent studies have demonstrated that a combination therapy composed of a selective HDAC6 inhibitor and a PD-1/PD-L1 inhibitor leads to significantly improved effects on tumor growth and antitumor immune response compared to a single therapy [31]. Therefore, the discovery of HDAC6 and immunotherapy-target dual inhibitors may provide a novel strategy for cancer treatment by taking advantage of both immunotherapeutic and epigenetic drugs.

Herein, we merged the common pharmacophores of HDAC inhibitors with the solvent exposure moieties of IDO1 inhibitors to design and synthesize a novel series of imidazothiazole-based hydroxamic acid derivatives as IDO1/HDAC6 dual inhibitors. Comprehensive evaluation identified the most potent inhibitor, **10e**, having potential IDO1 inhibitory activity and good selectivity against HDAC6. Subsequent in vivo experiments indicated that compound **10e** showed desirable in vivo antitumor efficacy in CT26 tumor-bearing mice.

## 2. Results

### 2.1. Rational Design of Novel IDO1 and HDAC6 Dual Inhibitors

**Figure 3:**
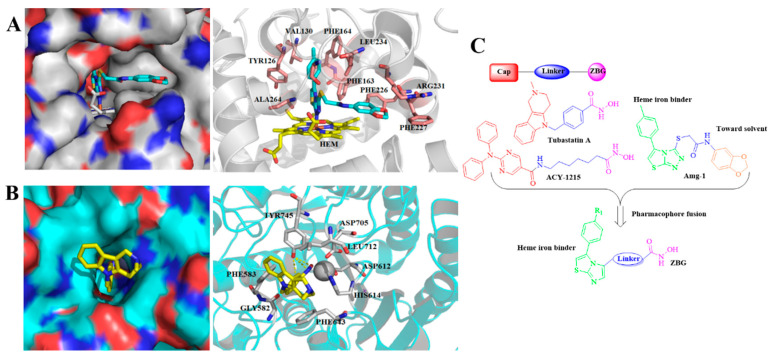
The design strategy of the IDO1/HDAC6 dual inhibitors. (**A**) The binding model of Amg-1 bound to IDO1 (PDB code 4PK5). The proteins are shown as 3D models. The carbon atoms of Amg-1 are shown in pink, and heme in yellow. (**B**) The binding model of Tubastatin A bound to HDAC6 (PDB code 6THV). The Tubastatin A is shown as sticks, with carbon atoms in cyan. (**C**) The pharmacophore fusion strategy for the design of the dual inhibitors.

The pharmacophore fusion strategy was carried out to design dual IDO1 and HDAC6 inhibitors. The imidazothiazole IDO1 inhibitor Amg-1 as well as the HDAC6 inhibitors Tubastatin A and ACY-1215 were used as the templates for the dual inhibitor design [15,32]. Generally, the classic pharmacophore model of HDAC inhibitors consists of a zinc-binding group (ZBG), a linker group, and a surface recognition cap group (Figure 3A) [33]. Among them, the cap group is a key point to gain subtype selectivity for HDAC inhibitors as it interacts with the surface of the wide basin around the entrance of the active site of the HDAC. On the other hand, according to the cocrystal structures of IDO1 and Amg-1 (Figure 3B), the nitrogen of the thiazolotriazole in Amg-1 directly coordinates with heme iron at the active site (pocket A) of IDO1, which has been identified as a crucial functional group for IDO1 inhibition [15]. The amide side chain of Amg-1, located at the expanded pocket B at the rim of the active site, could be modified without sacrificing IDO1 binding affinity. Thus, we fused the ZBG substructure into the IDO1 heme-binding imidazothiazole scaffold [14] via several different linkers (Figure 3C), and in this way, a series of novel IDO1/HDAC6 dual inhibitors were designed, synthesized, and biologically evaluated.

### 2.2. Chemistry

**Scheme 1:**
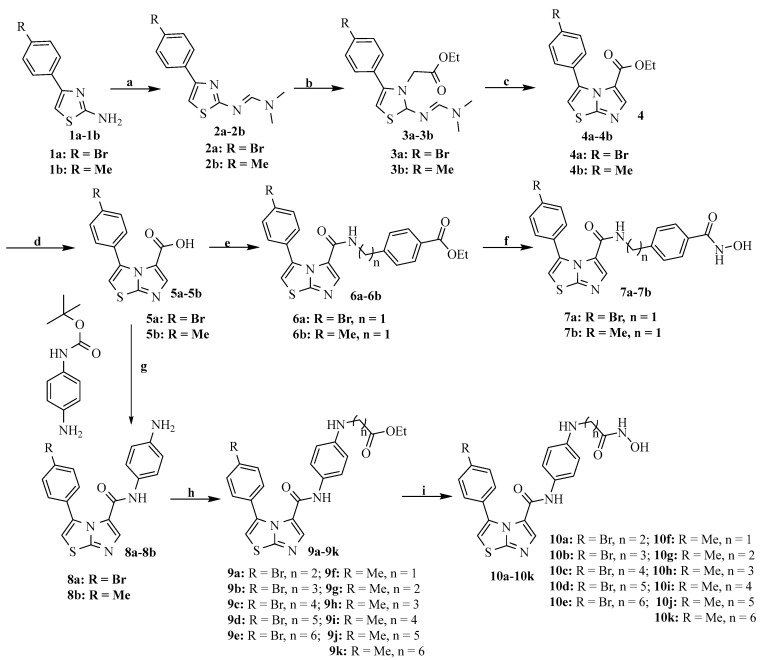
Synthetic routes of compounds **7a**, **7b**, and **10a**–**10k**. Reagents and conditions: (**a**) *N*,*N*-Dimethylformamide dimethyl acetal, DMF, 70 °C, 3 h; (**b**) Ethyl 2-bromoacetate, 80 °C, 12 h; (**c**) DBU, DMF, 80 °C, 9 h; (**d**) NaOH, THF/MeOH, rt, 12 h; (**e**) EDCI, HOBT, Et_3_N, CH_2_Cl_2_, 0 °C, rt, 12 h; (**f**) NH_2_OH·HCl, MeOH, 0 °C, 0.5 h; (**g**) EDCI, HOBT, Et_3_N, CH_2_Cl_2_, 0 °C, rt, 12 h; (**h**) DIPEA, DMF, 100 °C, 5–8 h; (**i**) NH_2_OH·HCl, MeOH, 0 °C, 0.5 h.

The detailed synthetic route to all the precursors for the target compounds (**7a** and **7b** and **10a**–**10k**) is outlined in Scheme 1. Treatment of the commercially available 2-amino-4-(4-bromine phenyl) thiazole (**1**) with *N*,*N*-dimethylformamide dimethylacetal gave compound **2**, which further reacted with ethyl bromoacetate to provide intermediate **3**. Imidazothiazole compound **4** was formed in the presence of DBU, and the subsequent treatment of intermediate **4** with an alkaline solution yielded imidazothiazole carboxylic acid compound **5**. The condensation of **5** with different amino-phenyl esters yielded ester **6**, which was subsequently reacted with a hydroxylamine methanol solution to give the target compounds **7a** and **7b**. The condensation of **5** with *N*-Boc-*p*-phenylenediamine followed by Boc deprotection yielded compound **8**, which reacted with commercially available bromo-alkanes under basic conditions to produce intermediate **9**. Finally, target compounds **10a**–**10k** were prepared using conditions similar to those used for compounds **7a** and **7b**.

### 2.3. In Vitro IDO1/HDAC6 Enzyme Inhibition

Initially, all the target compounds were assayed for their inhibitory activity against IDO1 and HDAC6 with the IDO1 inhibitor epacadostat and the HDAC inhibitor SAHA as the positive controls. As shown in Table 1, compound **7a** showed comparable HDAC6 inhibitory activity to SAHA, with 85.64% and 91.66% inhibition rates at 1 μM, respectively, but weaker IDO1 inhibitory activity than epacadostat. Generally, the linkers between the HDAC Zn-binding group and IDO1 heme-binding scaffold played an important role in enzyme inhibition. Compound **10a**, with a two-carbon linker, showed limited inhibitory potency against both targets, while compound **10d**, formed by extending the linker to a five-carbon chain, had significantly improved enzyme inhibition potency. The extension of the carbon chain resulted in increased activity against both targets. Specifically, compound **10e**, with a linker of six carbons, exhibited the most robust potency against HDAC6, with an inhibition rate of 90.45% at 1 μM, and was slightly less potent than SAHA. On the other hand, the substituent at the R position also played a role in the enzyme inhibitory activities. In general, the compounds with a bromine substituent displayed better activities than the methyl-substituted compounds. Compound **7a**, with a bromine substituent, was more potent than **7b**. Similarly, the replacement of the bromine group in compound **10e** with methyl (**10k**) resulted in decreased activity against both targets. Taken together, compound **10e**, with a linker length of six atoms and a bromine substituent, demonstrated good inhibitory capacity against both IDO1 and HDAC6.

### 2.4. HDAC Isoform Selectivity of Compounds ***7a***, ***10e***, and ***10k***

To explore the inhibitory activity toward other HDAC isoforms, the selected compounds **7a**, **10e**, and **10k** were further tested for their in vitro inhibitory activities against HDAC1, HDAC4, HDAC6, and HDAC11, with SAHA as the positive control. As shown in Table 2, all three compounds showed better activities against HDAC6 than HDAC1, HDAC4, and HDAC11. Compared with SAHA, all three selected compounds exhibited decreased inhibitory activities against HDAC1 and HDAC6, but showed a higher selectivity for HDAC6 than that of SAHA. Moreover, the HDAC6 inhibitory activity of compound **10e** (IC_50_ = 58.23 nM) was better than that of **7a** (IC_50_ = 84.44 nM). In particular, compound **10e** had a slightly weaker inhibitory activity, but its selectivity toward HDAC6 was 7-fold higher than that of SAHA.

### 2.5. Binding Modes of Compound ***10e*** with IDO1 and HDAC6

**Figure 4:**
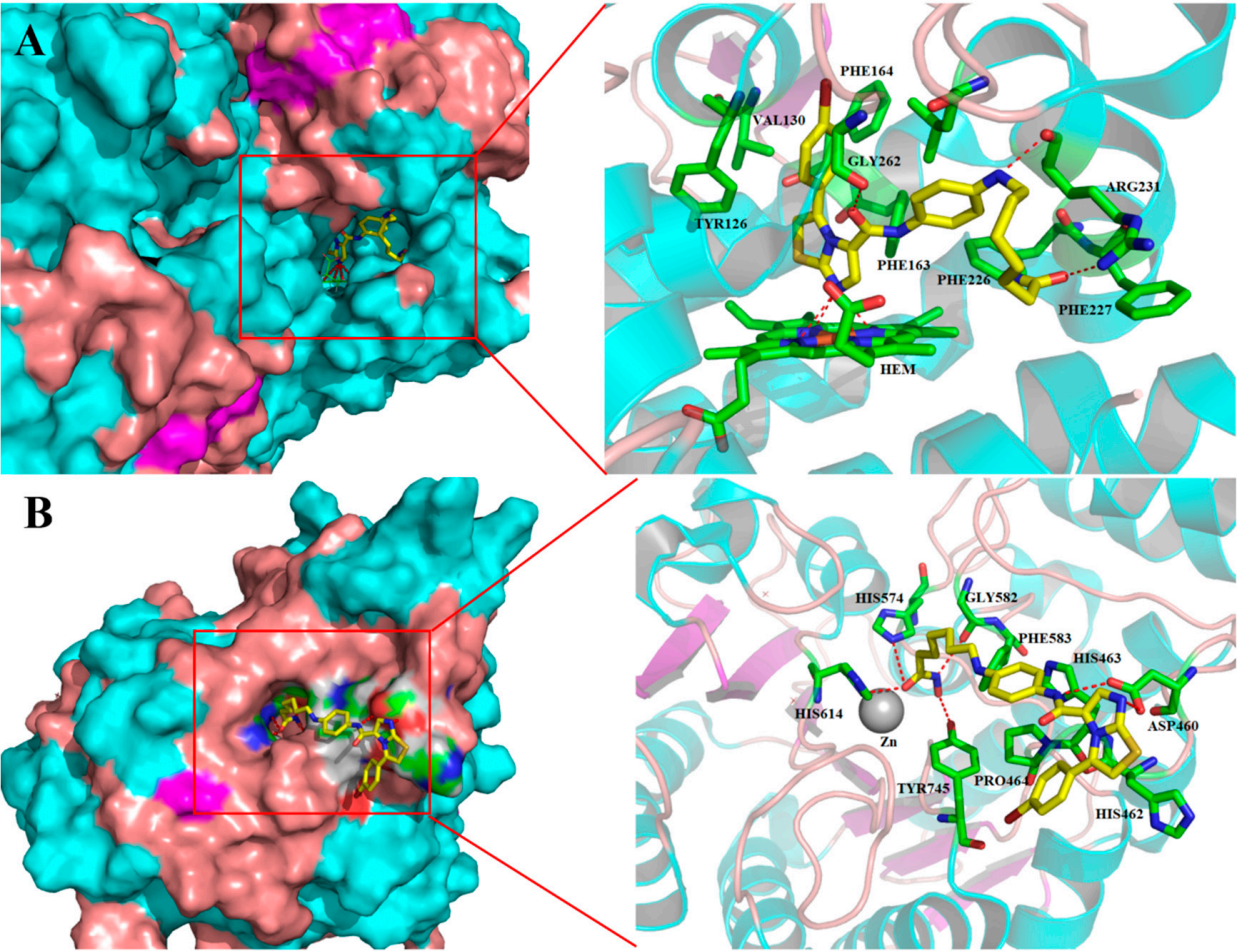
Proposed binding modes of compound **10e** (yellow) to IDO1 and HDAC6. (**A**) Compound **10e** docked into IDO1 (PDB ID: 4PK5). (**B**) Compound **10e** docked into HDAC6 (PDB ID: 6THV).

The above results confirm that compound **10e** acted as a dual IDO1/HDAC6 inhibitor. Next, molecular docking analysis was performed to uncover the possible binding modes of compound **10e** with IDO1 (PDB ID: 4PK5) [34] and HDAC6 (PDB ID: 6THV) [35], respectively, using SYBYL-X2.1 software. As shown in Figure 4A, compound **10e** perfectly fitted into the binding interface formed by IDO1 (docking score: 11.97), and the central imidazothiazole scaffold of **10e** directly bound to the heme iron and formed four hydrogen bonds with the porphyrin ring of heme, which contributed to its inhibitory activity against IDO1. The benzene ring on the imidazothiazole scaffold fitted into the hydrophobic pocket formed by the residues TYR126, VAL130, PHE163, PHE164, and LEU234. In addition, the π-π interactions were observed between the benzene ring and the key residues TYR126 and PHE164 of IDO1. Additionally, the carbonyl and amine moieties of the linker formed a hydrogen bond with the side chains of GLY262 and ARG231, respectively. In addition, the hydroxamic acid moiety also formed a stable hydrogen bond with key residue ARG231, enhancing its affinity. The binding mode of **10e** with HDAC6 (docking score: 9.60) is displayed in Figure 4B. As expected, the hydroxamic acid of **10e** coordinated with the catalytic zinc ion in the catalytic pocket and formed four hydrogen bonds with HIS614, HIS574, GLY582, and TYR745. Additionally, the amide part of the linker between the imidazothiazole scaffold and ZBG of compound **10e** formed a hydrogen bond with ASP460 at the rim of the hHDAC6 binding pocket. Specifically, the terminal imidazothiazole scaffold of **10e**, which also formed a hydrogen bond with ASP460, extended out of the binding site and was thus exposed to the solvent.

### 2.6. In Vitro Antiproliferative Activity of Selected Compounds

On the basis of the IDO1 and HDAC activities, we explored the proliferation inhibitory activities of the selected compounds against four solid tumor cells lines. The pan-HDAC SAHA was used as the positive control. The antiproliferative activities of selected compounds are shown in Table 3. As shown in Table 3, SAHA treatment generally resulted in strong antiproliferative effects, with IC_50_ values in the low micromolar range from 2.64 to 18.32 μM, whereas epacadostat was inactive against the four solid tumor cell lines. The data in Table 3 implied that all selected compounds and **5a** possessed weak cytotoxicity against the tested cell lines, which was much weaker than that of SAHA. Additionally, compound **10e** exhibited moderate antiproliferative activities against HCT-116 and SW480 cells, with IC_50_ values of 16.42 and 23.43 μM, respectively. It has been reported that IDO1 inhibitors do not destroy tumor cells directly. In addition, ricolinostat, an HDAC6 inhibitor with reduced class I HDAC inhibition, has low clinical activity when applied as monotherapy [36]. Noonepalle et al. showed that the selective HDAC6 inhibitor Suprastat was not cytotoxic up to 25 μM, while NextA began to induce cytotoxicity at a concentration of 10 μM [30]. Vögerl et al. also showed that selective HDAC6 inhibitors inhibited the proliferation of HUH7, MDA-MB-231, and T24 cells at concentrations of 10 and 100 μM after 72 h [37]. The possible reasons for the low cytotoxicity of **10e** may be the alteration of key cancer-related pathways, such as immune checkpoints and unfolded protein response. It is worth noting that compounds **10e** and **5a** showed low toxicity to human normal renal cells compared with SAHA (IC_50_ = 26.60 ± 4.31 μM). These findings further emphasized that **10e** was a selective HDAC6 inhibitor.

### 2.7. Inhibition of HDAC in HCT-116 Cells by Compound ***10e***

**Figure 5:**
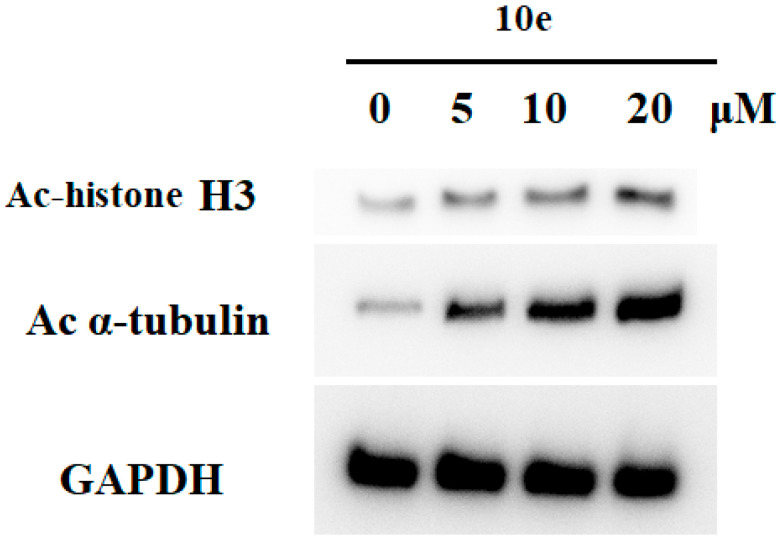
Western blots of Ac-α-tubulin and Ac-histone H3 after treatment of HCT-116 cells with compound **10e**. GAPDH was loading control.

Since HDACs regulate the acetylation status of histone and nonhistone proteins in cells, the acetylation levels of histone H3 (a known substrate for HDACs 1, 2, and 3) and α-tubulin (a known substrate for HDAC6) are frequently used as markers of cellular HDAC activity. To assess the potency and isoform selectivity of **10e** in cells, we performed a Western blot assay to investigate the in vitro inhibition of HDAC markers. HCT-116 cells were incubated with compound **10e** (5−20 μM) for 24 h, and the levels of acetylation of histone H3 and α-tubulin were detected by the Western blot. As shown in Figure 5, compound **10e** markedly increased Ac-α-tubulin levels in a concentration-dependent manner, and this feature was consistent with the signatory feature of the HDAC6 inhibitors. However, treatment with **10e** led to a slight increase in the levels of Ac-histone H3 at high concentrations of 20 μM, which was consistent with a previous report [38]. These results demonstrated that compound **10e** exhibited good HDAC6 potency and selectivity when tested in HCT-116 cells.

### 2.8. Cell Cycle Arrest

**Figure 6:**
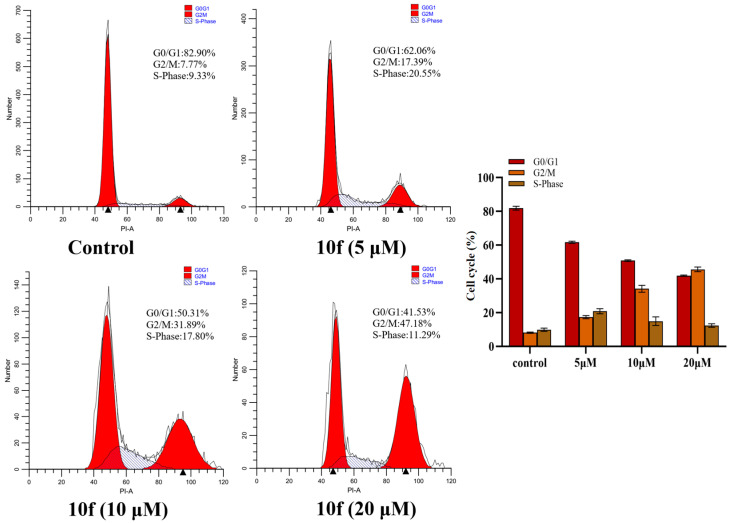
The cell cycle arrest effects of compound **10e** in HCT-116 cells. The cells were treated with 5, 10, and 20 μM of **10e** for 24 h, respectively, then stained with PI, and assessed by flow cytometry.

To assess the impact of compound **10e** on the cell cycle, HCT-116 cells were treated with different concentrations of compound **10e** (5 μM, 10 μM, and 20 μM) for 24 h, then stained with PI, and analyzed using flow cytometry. As displayed in Figure 6, compared with the control group (7.77%), the cell cycle analysis results revealed G2/M-phase cell cycle arrest, with increased cell population in the G2/M phase for **10e** (17.39%, 31.89%, and 47.18%). These results suggested that compound **10e** arrested the cell cycle of HCT-116 cells in the G2/M phase in a dose-dependent manner.

### 2.9. In Vivo Antitumor Effects

**Figure 7:**
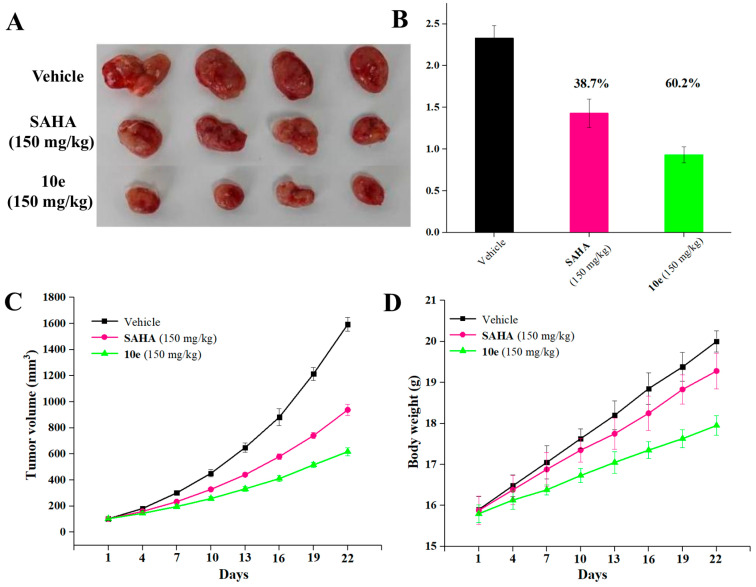
The in vivo antitumor activities of SAHA and compound **10e** against CT26 tumors in BALB/c mice. (**A**) Images of the harvested tumors from mice after the administration of SAHA (150 mg/kg) and **10e** (150 mg/kg) every 3 days for 3 weeks. (**B**) The tumor weights of the excised tumors of each group. (**C**) The tumor volume of the mice in each group during the observation period. (**D**) The body weight of the mice in each group at the end of the observation period. These data are presented as means ± SD. *p* < 0.05 (versus the vehicle control group).

**Figure 8:**
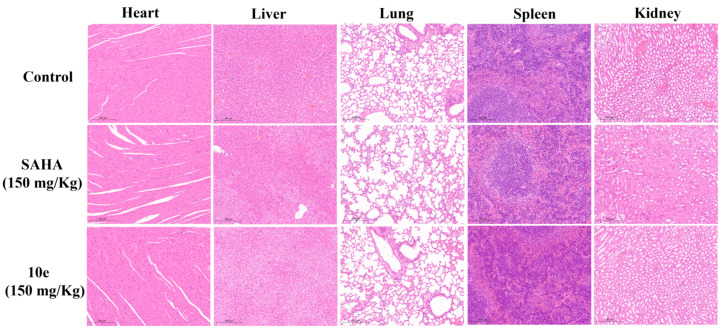
Histopathological changes in major organs dissected from tumor-bearing mice after **10e** treatment by H&E staining.

To further investigate the therapeutic capacity of compound **10e**, its in vivo antitumor efficacy was evaluated in a CT26 tumor-bearing BALB/c mice model. After the average tumor volume reached 100 mm^3^ in each group, SAHA (150 mg/kg) and compound **10e** (150 mg/kg) were administered intraperitoneally (ip) every 3 days for 21 consecutive days. As depicted in Figure 7A, compound **10e** exhibited significant in vivo activity in inhibiting tumor growth compared with the vehicle group. At a dose of 150 mg/kg, compound **10e** achieved a tumor growth inhibition (TGI) value of 60.2% (Figure 7B), which was more potent than SAHA (TGI = 38.7%). At the treatment end point, compound **10e** treatment resulted in significantly smaller tumor volumes compared to the control group. The relative tumor volume growth rate (T/C) of **10e** was 38.32% at 150 mg/kg, which was better than that of SAHA (58.97% at 150 mg/kg) (Figure 7C). Moreover, both **10e** (150 mg/kg) and SAHA treatments displayed no obvious influence on the body weight of the mice in comparison with the vehicle group (Figure 7D). Additionally, the cellular morphology of the liver, spleen, and kidney in the H&E staining images revealed that no obvious histological differences were observed in the compound **10e**-treated group (Figure 8), indicating its low toxicity toward normal organs. These results indicated that compound **10e** effectively inhibited the growth of tumors with low general toxicity.

## 3. Materials and Methods

### 3.1. General Information

All raw materials, reagents, and solvents were analytical-grade and commercially available. Silica gel (200–300 mesh) was used for column chromatography. ^1^H NMR and ^13^C NMR spectra were recorded in DMSO-*d_6_* on a 400 MHz (^1^H, 400 MHz; ^13^C, 101 MHz) Bruker spectrometer (AVANCE NEO 400M) or 600 MHz (^1^H, 600 MHz; ^13^C, 151 MHz) Bruker spectrometer (AVANCE NEO 600M) with TMS as an internal reference. High resolution mass spectra (HR-MS) were evaluated on an Agilent 1290–6545 UHPLC-QTOF mass spectrometers (Agilent Technologies, Santa Clara, CA, USA).

### 3.2. General Procedure for Preparation of Compounds ***7a***, ***7b***, and ***10a***–***10k***

Equimolar ratios of 2-amino-4-(4-bromophenyl)thiazole or 2-amino-4-(4-tolyl)thiazole (6.00 g, 0.02 mol) with *N*,*N*-dimethylformamide dimethyl acetal (2.38 g, 0.02 mol) were refluxed with stirring in DMF (30 mL) at 70 °C for 3 h. Washings with water and ethyl acetate resulted in the concentration of compound **2**. Compound **2** (5.9 g, 0.02 mol) was refluxed with ethyl bromoacetate (6.68 g, 0.04 mol) with stirring at 80 °C for 12 h. The resulting precipitate was filtered and washed with ethyl acetate to give the pale yellow bromide **3**. Bromide **3** (5.37 g, 0.01 mol) was condensed and refluxed with DBU (0.03 mol) in DMF (30 mL) for 9 h and concentrated under reduced pressure to give compound **4**. Compound **4** (5.37 g, 0.01 mol) was stirred in a mixture of tetrahydrofuran and methanol (30 mL) with 1N NaOH (22.5 mL) overnight, and the reaction was monitored by TLC. The reaction was concentrated under reduced pressure and acidified with 1N HCl to pH = 4; the precipitate was filtered and washed with water to obtain the white solid intermediate **5**.

Compound **2a**: HR-MS (*m*/*z*): calcd for C_12_H_12_BrN_3_S [M + H]^+^: 310.0013; found: 310.0033.

Compound **3a**: HR-MS (*m*/*z*): calcd for C_16_H_20_BrN_3_O_2_S [M + H]^+^: 398.0538; found: 398.0375.

Compound **4a**: HR-MS (*m*/*z*): calcd for C_14_H_11_BrN_2_O_2_S [M + H]^+^: 350.9803; found: 350.9818.

Intermediate **5a.** ^1^H NMR (400 MHz, DMSO-*d*_6_) *δ* 12.57 (s, 1H), 7.92 (d, *J* = 1.2 Hz, 1H), 7.63 − 7.60 (m, 2H), 7.44 − 7.41 (m, 3H). ^13^C NMR (101 MHz, DMSO-*d*_6_) *δ* 159.21, 154.34, 142.17, 133.19, 130.66, 130.27, 130.12, 122.06, 120.77, 113.79. HR-MS (*m/z*) (ESI): calcd for C_12_H_7_BrN_2_O_2_S [M + H]^+^: 322.9490; found: 322.9492.

For the preparation of compounds **7a, 7b**, and **10a**–**10k**, intermediate **5** (3.22 g, 0.01 mol) was mixed with EDCI (2.30 g, 0.012 mol), HOBT (1.62g, 0.012 mol), and Et_3_N (2.77 mL, 0.02 mol) in DCM solution at 0 °C, stirred for several minutes, and then ethyl 4-amino-benzoate or ethyl 4-aminomethyl-benzoate (1.98 g, 0.012 mol) was added and stirred overnight at room temperature. Silica gel chromatography (DCM/MeOH = 20: 1 *v*/*v*) was used to purify compound **6**. Compound **6** was mixed with hydroxylamine hydrochloride methanol solution, stirred at 0 °C for 0.5 h, and then adjusted to pH = 7 with 1N HCl, filtered, and TCL purified to obtain compounds **7a** and **7b**. Compound **8** was synthesized in the same way as compound **6**, and was first synthesized into compounds with Boc protecting groups and then deprotected with a 20% TFA/DCM solution to obtain compound **8**. Compound **8** (4.12 g, 0.01 mol) was mixed with ethyl esters (0.01 mol) and DIPEA (4.85 mL, 0.03 mol) in DMF (15 mL) solution under stirring and reflux at 100 °C for 5–8 h to obtain compound **9**. Compound **9** was reacted with hydroxylamine hydrochloride methanol solution at 0 °C and purified by TCL to obtain compounds **10a**–**10k**.

Compound **6a**: HR-MS (*m*/*z*): calcd for C_22_H_18_BrN_3_O_3_S [M + H]^+^: 484.0330; found: 484.0325.

Compound **8a**: HR-MS (*m*/*z*): calcd for C_18_H_13_BrN_4_OS [M + H]^+^: 413.0071; found: 413.0095.

Compound **9e**: HR-MS (*m*/*z*): calcd for C_27_H_29_BrN_4_O_3_S [M + H]^+^: 569.1222; found: 569.1220.

*3-(4-Bromophenyl)-N-[4-(hydroxycarbamoyl)benzyl]imidazo[2,1-b]thiazole-5-carboxamide* (**7a**)**.** Yield: 41.55%, as a white solid. ^1^H NMR (400 MHz, DMSO-*d*_6_) δ 11.21 (s, 1H), 9.00 (t, *J* = 6.1 Hz, 1H), 7.81 − 7.79 (m, 1H), 7.76 − 7.73 (m, 2H), 7.49 − 7.46 (m, 2H), 7.40 (d, *J* = 1.1 Hz, 1H), 7.35 − 7.33 (m, 2H), 7.31 (d, *J* = 8.2 Hz, 2H), 4.27 (d, *J* = 6.0 Hz, 2H). ^13^C NMR (101 MHz, DMSO-*d*_6_) δ 158.35, 152.34, 142.69, 137.43, 132.67, 131.29, 130.80, 129.97, 129.19, 127.10, 126.88, 123.57, 121.91, 113.00, 41.88. HR-MS (*m/z*) (ESI): calcd for C_20_H_15_BrN_4_O_3_S [M + Na]^+^: 494.9926; found: 494.9918.

*N-[4-(hydroxycarbamoyl)benzyl]-3-(p-tolyl)imidazo[2,1-b]thiazole-5-carboxamide* (**7b**). Yield: 40.19%, as a white solid. ^1^H NMR (600 MHz, DMSO-*d*_6_) δ 11.22 (s, 1H), 9.04 (s, 1H), 8.95 (t, *J* = 6.1 Hz, 1H), 7.73 (d, *J* = 7.8 Hz, 3H), 7.30 (d, *J* = 8.0 Hz, 2H), 7.28 − 7.25 (m, 3H), 7.09 (d, *J* = 7.7 Hz, 2H), 4.24 (d, *J* = 6.1 Hz, 2H), 2.31 (s, 3H). ^13^C NMR (151 MHz, DMSO-*d*_6_) δ 164.01, 158.47, 152.13, 142.67, 138.15, 137.18, 133.85, 131.24, 128.49, 127.71, 127.21, 126.80, 123.71, 111.44, 41.96, 20.91. HR-MS (*m*/*z*) (ESI): calcd for C_21_H_18_N_4_O_3_S [M + H]^+^: 407.1180; found: 407.1171.

*3-(4-Bromophenyl)-N-{4-[(3-(hydroxyamino)-3-oxopropyl)amino]phenyl}imidazo[2,1-b]thiazole-5-carboxamide* (**10a**). Yield: 33.69%, as a white solid. ^1^H NMR (400 MHz, DMSO-*d*_6_) δ 10.46 (s, 1H), 9.92 (s, 1H), 8.79 (s, 1H), 7.84 (s, 1H), 7.57 (d, *J* = 8.4 Hz, 2H), 7.42 (s, 1H), 7.39 (d, *J* = 8.4 Hz, 2H), 7.13 (d, *J* = 8.8 Hz, 2H), 6.48 (d, *J* = 8.8 Hz, 2H), 5.46 (t, *J* = 5.9 Hz, 1H), 3.21 (d, *J* = 6.4 Hz, 2H), 2.22 (t, *J* = 7.1 Hz, 2H). ^13^C NMR (101 MHz, DMSO-*d*_6_) δ 167.73, 156.04, 152.09, 145.23, 137.51, 132.73, 131.00, 129.91, 129.21, 127.68, 124.17, 122.08, 121.77, 112.94, 111.84, 48.66, 32.34. HR-MS (*m/z*) (ESI): calcd for C_21_H_18_BrN_5_O_3_S [M + H]^+^: 500.0394; found: 500.0364.

*3-(4-Bromophenyl)-N-{4-[(4-(hydroxyamino)-4-oxobutyl)amino)phenyl}imidazo[2,1-b]thiazole-5-carboxamide* (**10b**). Yield: 31.12%, as a white solid. ^1^H NMR (400 MHz, DMSO-*d*_6_) δ 10.38 (s, 1H), 9.88 (s, 1H), 8.70 (s, 1H), 7.82 (s, 1H), 7.57 (d, *J* = 8.5 Hz, 2H), 7.42 (d, *J* = 1.1 Hz, 1H), 7.39 (d, *J* = 8.5 Hz, 2H), 7.11 (d, *J* = 8.9 Hz, 2H), 6.45 (d, *J* = 8.9 Hz, 2H), 5.46 (s, 1H), 2.94 (s, 2H), 2.04 (t, *J* = 7.4 Hz, 2H), 1.73 (t, *J* = 7.1 Hz, 2H). ^13^C NMR (101 MHz, DMSO-*d*_6_) δ 169.04, 155.99, 151.99, 145.63, 137.39, 132.71, 130.97, 129.87, 129.19, 127.38, 124.18, 122.04, 121.71, 112.85, 111.64, 42.67, 29.98, 24.80. HR-MS (*m/z*) (ESI): calcd for C_22_H_20_BrN_5_O_3_S [M + H]^+^: 516.0530; found: 516.0522.

*3-(4-Bromophenyl)-N-{4-[(5-(hydroxyamino)-5-oxopentyl)amino)phenyl}imidazo[2,1-b]thiazole-5-carboxamide* (**10c**). Yield: 37.11%, as a white solid. ^1^H NMR (400 MHz, DMSO-*d*_6_) δ 10.35 (s, 1H), 9.87 (s, 1H), 8.68 (s, 1H), 7.82 (s, 1H), 7.58 − 7.56 (m, 2H), 7.42 (d, *J* = 1.1 Hz, 1H), 7.41 − 7.38 (m, 2H), 7.10 (d, *J* = 8.9 Hz, 2H), 6.46 (d, *J* = 8.9 Hz, 2H), 5.42 (s, 1H), 2.95 (t, *J* = 6.8 Hz, 2H), 1.97 (t, *J* = 7.1 Hz, 2H), 1.56 (d, *J* = 7.8 Hz, 2H), 1.49 (t, *J* = 6.9 Hz, 2H). ^13^C NMR (101 MHz, DMSO-*d*_6_) δ 169.00, 155.97, 151.96, 145.76, 137.42, 132.69, 130.94, 129.87, 129.15, 127.24, 124.16, 121.99, 121.67, 112.86, 111.55, 42.74, 32.05, 28.23, 22.87. HR-MS (*m/z*) (ESI): calcd for C_23_H_22_BrN_5_O_3_S [M + H]^+^: 530.0686; found: 530.0682.

*3-(4-Bromophenyl)-N-{4-[6-(hydroxyamino)-6-oxohexyl)amino]phenyl}imidazo[2,1-b]thiazole-5-carboxamide* (**10d**). Yield: 29.54%, as a white solid. ^1^H NMR (400 MHz, DMSO-*d*_6_) δ 10.35 (s, 1H), 9.87 (s, 1H), 8.68 (d, *J* = 1.8 Hz, 1H), 7.82 (s, 1H), 7.58 − 7.56 (m, 2H), 7.42 (d, *J* = 1.3 Hz, 1H), 7.40 − 7.38 (m, 2H), 7.10 (d, *J* = 8.9 Hz, 2H), 6.46 − 6.44 (m, 2H), 5.41 (s, 1H), 2.93 (t, *J* = 7.1 Hz, 2H), 1.95 (t, *J* = 7.4 Hz, 2H), 1.51 (t, *J* = 7.6 Hz, 4H), 1.33 − 1.29 (m, 2H). ^13^C NMR (101 MHz, DMSO-*d*_6_) δ 169.06, 155.97, 151.94, 145.79, 137.40, 132.68, 130.94, 129.89, 129.17, 127.28, 124.19, 122.00, 121.70, 112.83, 111.52, 43.02, 32.29, 28.47, 26.33, 25.05. HR-MS (*m/z*) (ESI): calcd for C_24_H_24_BrN_5_O_3_S [M + H]^+^: 544.0843; found: 544.0846.

*3-(4-Bromophenyl)-N-{4-[(7-(hydroxyamino)-7-oxoheptyl)amino]phenyl}imidazo[2,1-b]thiazole-5-carboxamide* (**10e**). Yield: 30.97%, as a white solid. ^1^H NMR (400 MHz, DMSO-*d*_6_) δ 10.36 (s, 1H), 9.87 (s, 1H), 8.68 (s, 1H), 7.82 (s, 1H), 7.56 (d, *J* = 8.1 Hz, 2H), 7.43 (s, 1H), 7.39 (d, *J* = 8.3 Hz, 2H), 7.10 (d, *J* = 8.5 Hz, 2H), 6.45 (d, *J* = 8.6 Hz, 2H), 5.37 (s, 1H), 2.93 (d, *J* = 5.4 Hz, 2H), 1.95 (t, *J* = 7.3 Hz, 2H), 1.50 (d, *J* = 6.4 Hz, 4H), 1.29 (dd, *J* = 24.0, 7.9 Hz, 4H). ^13^C NMR (101 MHz, DMSO-*d*_6_) δ 169.07, 155.97, 151.95, 145.81, 137.43, 132.68, 130.93, 129.87, 129.15, 127.25, 124.17, 121.99, 121.70, 112.85, 111.52, 43.09, 32.19, 28.62, 28.47, 26.44, 25.15. HR-MS (*m/z*) (ESI): calcd for C_25_H_26_BrN_5_O_3_S [M + H]^+^: 558.0999; found: 558.0971.

*N-{4-[(2-(hydroxyamino)-2-oxoethyl)amino]phenyl}-3-(p-tolyl)imidazo[2,1-b]thiazole-5-carboxamide* (**10f**). Yield: 34.86%, as a white solid. ^1^H NMR (400 MHz, DMSO-*d*_6_) δ 10.59 (s, 1H), 9.90 (s, 1H), 8.83 (s, 1H), 7.77 (s, 1H), 7.34 − 7.32 (m, 2H), 7.31 (d, *J* = 1.1 Hz, 1H), 7.17 (d, *J* = 8.1 Hz, 2H), 7.09 (d, *J* = 8.9 Hz, 2H), 6.47 − 6.43 (m, 2H), 5.74 (t, *J* = 6.4 Hz, 1H), 3.53 (d, *J* = 6.3 Hz, 2H), 2.29 (s, 3H). ^13^C NMR (101 MHz, DMSO-*d*_6_) δ 167.08, 156.14, 151.86, 144.91, 138.36, 137.25, 133.89, 128.65, 128.21, 127.65, 126.85, 124.27, 121.53, 112.05, 111.40, 44.69, 20.93. HR-MS (*m/z*) (ESI): calcd for C_21_H_19_N_5_O_3_S [M + Na]^+^: 444.1107; found: 444.1104.

*N-{4-[(3-(hydroxyamino)-3-oxopropyl)amino]phenyl}-3-(p-tolyl)imidazo[2,1-b]thiazole-5-carboxamide* (**10g**). Yield: 35.23%, as a white solid. ^1^H NMR (400 MHz, DMSO-*d*_6_) δ 10.54 (s, 1H), 9.85 (s, 1H), 8.79 (s, 1H), 7.76 (s, 1H), 7.35 − 7.32 (m, 2H), 7.29 (d, *J* = 1.1 Hz, 1H), 7.17 (d, *J* = 8.3 Hz, 2H), 7.09 (d, *J* = 8.8 Hz, 2H), 6.46 (d, *J* = 8.9 Hz, 2H), 5.68 (t, *J* = 6.3 Hz, 1H), 4.08 (q, *J* = 5.3 Hz, 1H), 3.54 (d, *J* = 6.1 Hz, 1H), 3.17 (d, *J* = 5.3 Hz, 2H), 2.29 (s, 3H). ^13^C NMR (101 MHz, DMSO-*d*_6_) δ 167.00, 156.11, 151.77, 144.86, 138.29, 137.15, 133.85, 128.57, 128.18, 127.60, 126.82, 124.25, 121.49, 112.02, 111.26, 48.56, 44.69, 20.85. HR-MS (*m/z*) (ESI): calcd for C_22_H_21_N_5_O_3_S [M + H]^+^: 436.1445; found: 436.1437.

*N-{4-[(4-(hydroxyamino)-4-oxobutyl)amino]phenyl}-3-(p-tolyl)imidazo[2,1-b]thiazole-5-carboxamide* (**10h**). Yield: 34.22%, as a white solid. ^1^H NMR (400 MHz, DMSO-*d*_6_) δ 10.36 (s, 1H), 9.80 (s, 1H), 8.68 (s, 1H), 7.75 (s, 1H), 7.35 − 7.32 (m, 2H), 7.28 (d, *J* = 1.1 Hz, 1H), 7.16 (d, *J* = 8.1 Hz, 2H), 7.08 (d, *J* = 8.9 Hz, 2H), 6.45 − 6.42 (m, 2H), 5.41 (t, *J* = 5.6 Hz, 1H), 2.94 (d, *J* = 6.4 Hz, 2H), 2.28 (s, 3H), 2.04 (t, *J* = 7.4 Hz, 2H), 1.77 − 1.70 (m, 2H). ^13^C NMR (101 MHz, DMSO-*d*_6_) δ 168.99, 156.06, 151.70, 145.53, 138.29, 137.05, 133.87, 128.59, 127.59, 127.46, 126.85, 124.33, 121.63, 111.52, 111.22, 42.66, 29.93, 24.78, 20.85. HR-MS (*m/z*) (ESI): calcd for C_23_H_23_N_5_O_3_S [M + H]^+^: 450.1602; found: 450.1593.

*N-{4-[(5-(hydroxyamino)-5-oxopentyl)amino]phenyl}-3-(p-tolyl)imidazo[2,1-b]thiazole-5-carboxamide* (**10i**). Yield: 28.13%, as a white solid. ^1^H NMR (400 MHz, DMSO-*d*_6_) δ 10.35 (s, 1H), 9.80 (s, 1H), 8.66 (s, 1H), 7.75 (s, 1H), 7.35 − 7.32 (m, 2H), 7.28 (d, *J* = 1.0 Hz, 1H), 7.16 (d, *J* = 8.1 Hz, 2H), 7.07 (d, *J* = 8.9 Hz, 2H), 6.45 − 6.42 (m, 2H), 5.36 (t, *J* = 5.7 Hz, 1H), 2.94 (q, *J* = 6.6 Hz, 2H), 2.28 (s, 3H), 1.98 (t, *J* = 7.3 Hz, 2H), 1.61 − 1.54 (m, 2H), 1.52 − 1.45 (m, 2H). ^13^C NMR (101 MHz, DMSO-*d*_6_) δ 169.00, 156.07, 151.70, 145.66, 138.29, 137.04, 133.87, 128.59, 127.60, 127.33, 126.85, 124.35, 121.66, 111.49, 111.23, 42.74, 32.04, 28.24, 22.84, 20.85. HR-MS (*m/z*) (ESI): calcd for C_24_H_25_N_5_O_3_S [M + H]^+^: 464.1758; found: 464.1745.

*N-{4-[(6-(hydroxyamino)-6-oxohexyl)amino]phenyl}-3-(p-tolyl)imidazo[2,1-b]thiazole-5-carboxamide* (**10j**). Yield: 38.31%, as a white solid. ^1^H NMR (400 MHz, DMSO-*d*_6_) δ 10.34 (s, 1H), 9.81 (s, 1H), 8.66 (s, 1H), 7.75 (s, 1H), 7.33 (d, *J* = 8.1 Hz, 2H), 7.29 (d, *J* = 1.3 Hz, 1H), 7.16 (d, *J* = 8.0 Hz, 2H), 7.07 (d, *J* = 8.9 Hz, 2H), 6.44 − 6.42 (m, 2H), 5.35 (t, *J* = 5.7 Hz, 1H), 2.93 (d, *J* = 6.0 Hz, 2H), 2.28 (s, 3H), 1.95 (t, *J* = 7.4 Hz, 2H), 1.54 − 1.48 (m, 4H), 1.34 (d, *J* = 6.4 Hz, 2H). ^13^C NMR (101 MHz, DMSO-*d*_6_) δ 169.06, 156.06, 151.71, 145.72, 138.31, 137.08, 133.87, 128.62, 127.62, 127.34, 126.86, 124.35, 121.64, 111.47, 111.28, 43.01, 32.29, 28.47, 26.32, 25.03, 20.89. HR-MS (*m/z*) (ESI): calcd for C_25_H_27_N_5_O_3_S [M + H]^+^: 478.1915; found: 478.1900.

*N-{4-[(7-(hydroxyamino)-7-oxoheptyl)amino]phenyl}-3-(p-tolyl)imidazo[2,1-b]thiazole-5-carboxamide* (**10k**). Yield: 26.79%, as a white solid. ^1^H NMR (400 MHz, DMSO-*d*_6_) δ 10.34 (s, 1H), 9.82 (s, 1H), 8.67 (s, 1H), 7.76 (s, 1H), 7.33 (d, *J* = 8.0 Hz, 2H), 7.29 (s, 1H), 7.16 (d, *J* = 8.1 Hz, 2H), 7.07 (d, *J* = 8.8 Hz, 2H), 6.43 (d, *J* = 8.9 Hz, 2H), 5.36 (t, *J* = 5.6 Hz, 1H), 2.93 (q, *J* = 6.7 Hz, 2H), 2.28 (s, 3H), 1.94 (t, *J* = 7.4 Hz, 2H), 1.53 − 1.46 (m, 4H), 1.38 − 1.26 (m, 4H). ^13^C NMR (101 MHz, DMSO-*d*_6_) δ 169.10, 156.06, 151.72, 145.74, 138.31, 137.09, 133.88, 128.63, 127.62, 127.33, 126.86, 124.36, 121.64, 111.47, 111.29, 43.08, 32.24, 28.62, 28.48, 26.43, 25.14, 20.89. HR-MS (*m/z*) (ESI): calcd for C_26_H_29_N_5_O_3_S [M + H]^+^: 492.2071; found: 492.2072.

### 3.3. Biological Assays

The biological experimental procedures, including the HDAC inhibitory assay, IDO1 inhibitory activity assay, wound healing assay, SPR assay, antiproliferative activity, molecular docking, cell cycle analysis, Western blot, and in vivo antitumor efficacy analyses were carried out according to our previous work [34,39,40,41] and are described in the Supplementary Information (SI).

## 4. Conclusions

In summary, a series of novel imidazothiazole hydroxamic acid derivatives were designed and synthesized as IDO1/HDAC6 dual inhibitors. Structure−activity relationship (SAR) exploration revealed several compounds as potent and selective IDO1/HDAC6 dual inhibitors, with IC_50_ values at the level of submicromolar concentrations. Particularly, the compound *3-(4-Bromophenyl)-N-{4-[(7-(hydroxyamino)-7-oxoheptyl)amino]phenyl}imidazo[2,1-b]thiazole-5-carboxamide* (**10e**) was identified as a potent IDO1/HDAC6 dual inhibitor with superior HDAC6 inhibitory capacities to HDAC1, HDAC4, and HDAC11 as well. The HDAC6 selectivity profile of **10e** was further supported by α-tubulin hyperacetylation. Docking studies illustrated the possible conformation of compound **10e** binding to IDO1 and HDAC6. Dual inhibitor **10e** displayed moderate antiproliferative activity against HCT-116 and SW480 cells, with IC_50_ values of 16.42 μM and 23.43 μM, respectively. Compound **10e** was found to arrest the cell cycle at the G2/M phase in HCT-116 cells. Importantly, compound **10e** exhibited good in vivo antitumor efficacy with no significant toxicity in a CT26 tumor-bearing BALB/c mice model. Consequently, these findings suggest that compound **10e** is a potential IDO1/HDAC6 dual inhibitor with potent anticancer activity.

## Figures and Tables

**Table 1:** In vitro enzyme inhibitory activity of compounds **7a**, **7b**, and **10a**–**10k** against IDO1 and HDAC6.

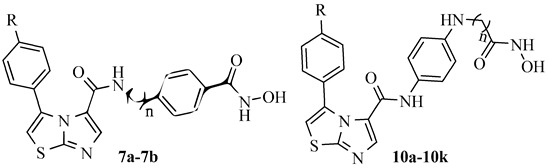				
Compd.	R	n	IDO1 IC_50_ (μM) ^a^	HDAC6 Inhibition Rate at 1 μM
**7a**	Br	1	0.53 ± 0.11	85.64%
**7b**	Me	1	0.96 ± 0.13	47.95%
**10a**	Br	2	1.87 ± 0.06	22.44%
**10b**	Br	3	1.06 ± 0.02	25.24%
**10c**	Br	4	0.34 ± 0.09	19.37%
**10d**	Br	5	0.12 ± 0.01	46.16%
**10e**	Br	6	0.086 ± 0.02	90.45%
**10f**	Me	1	6.16 ± 0.83	28.83%
**10g**	Me	2	2.07 ± 0.29	31.25%
**10h**	Me	3	1.78 ± 0.47	33.66%
**10i**	Me	4	0.59 ± 0.11	35.49%
**10j**	Me	5	0.38 ± 0.05	40.23%
**10k**	Me	6	0.16 ± 0.03	70.69%
Epacadostat	-	-	0.064 ± 0.01	-
SAHA	-	-	-	91.66%
**5a**	-	-	6.52 ± 0.45	-

^a^ IC_50_ values are the means ± S.D. of three independent experiments.

**Table 2:** Inhibition activity of selected compounds against HDAC subtypes.

	IC_50_ (nM) ^a^				
Compd.	HDAC1	HDAC4	HDAC6	HDAC11	SI ^c^
**7a**	2449	31.53% ^b^	84.44	6064.00	29.00
**10e**	1078	9.58%	58.23	24.37%	18.51
**10k**	1188	9.68%	390.70	29.77%	3.04
SAHA	90.87	33,911	37.17	52,903.00	2.44

^a^ IC_50_ performed using 10-dose IC_50_ mode with 3-fold serial dilution beginning from 10 μM. ^b^ % inhibition rate at 10 μM. ^c^ Selectivity Index (SI) = HDAC1 IC_50_ value/HDAC6 IC_50_ value.

**Table 3:** In vitro antiproliferative activity of selected compounds.

	IC_50_ (μM) ^a^				
Compd.	HCT-116	SW480	MDA-MB-231	MCF-7	HK2
**7a**	>80	>80	>80	>80	-
**10d**	24.43 ± 4.78	>80	>80	52.94 ± 6.95	-
**10e**	16.42 ± 3.76	23.43 ± 3.86	43.74 ± 1.89	36.46 ± 5.45	>80
**10j**	>80	>80	>80	>80	-
**10k**	>80	>80	>80	>80	-
SAHA	2.64 ± 4.26	4.32 ± 2.26	18.32 ± 5.61	6.32 ± 0.88	26.60 ± 4.31
Epacadostat	>80	>80	>80	>80	-
**5a**	>80	>80	>80	>80	>80

^a^ IC_50_ values are the means ± S.D. of three separate experiments.

## Data Availability

The data presented in this study are available on request from the authors.

## Supplementary Materials

The following supporting information can be downloaded at: https://www.mdpi.com/article/10.3390/molecules30122508/s1. Biological assays and Figures S1–S48. ^1^H-NMR, ^13^C-NMR, and HRMS spectra of all new compounds [15,35,42,43,44].
