# Supplementary material for: Discovery of Novel Imidazothiazole-Based Hydroxamic Acid Derivatives as Potent Indoleamine 2,3-Dioxygenase 1 and Histone Deacetylase 6 Dual Inhibitors
Source: Molecules. 2025 Jun 7;30(12):2508. doi: 10.3390/molecules30122508 (PMC12195740; doi:10.3390/molecules30122508)
Supplement: Supplementary file 1 [file molecules-30-02508-s001.zip › molecules-3655143-supplementary.pdf]

Section S1. Biological assays

Section S 1.1. HDAC6 enzyme inhibitory activity assay

Preparation of Master Mix. Trichostatin A in DMSO, Fluorogenic HDAC substrate 3, and HDAC6 human recombinant enzyme were diluted to 20 μM, 200 μM, and 7 ng/uL, respectively, using HDAC Assay Buffer.BSA was dissolved in 1 mL of distilled water to make a BSA solution at a concentration of 1 mg/mL. A certain amount of Fluorogenic HDAC substrate 3, BSA and HDAC Assay Buffer were mixed well to make a master mix, and 40 μL of the master mix was added to each well of a 96-well plate. In the drug administration group, 5 μL of Trichostatin A in DMSO and HDAC6 human recombinant enzyme were added to each well. 5 μL of HDAC Assay Buffer and HDAC6 human recombinant enzyme were added to each well of the blank group, and incubated for 30 min at 37 °C. Finally, 50 μL of undiluted HDAC substrate 3, BSA and HDAC Assay Buffer were added to each well. 50 μL of 2x HDAC Developer was added to each well and incubated at room temperature for 15 min. The samples were read in an enzyme marker with an excitation wavelength in the range of 350-380 nm and an emission light in the range of 440-460 nm. The values of fluorescence intensities were read and the inhibition rate and IC_{50} were calculated.

Section S 1.2 IDO1 inhibition assays

IDO1 inhibition assays were carried out according to the manufacturers' procedures, as described previously [42]. The standard assay mixture (100 μL) contained 50 mM of potassium phosphate buffer (pH = 6.5), 10 mM ascorbate, 10 μM methylene blue, 100 μg/mL catalase, 1 mM L-tryptophan and 10 μg/mL of rhIDO1. The plate was incubated at 37 °C in a dark environment, and reactions were terminated after 60 min by the addition of 50 μL of fluorogenic developer solution and further incubated at 45 °C in dark for 3 h. Then, the plate was allowed to cool to room temperature for at least 1 h. After centrifugation at 1500g for 5 min at 20 °C, the fluorescence intensity (Ex/Em = 402/488 nm) was measured with a Fluoroskan Ascent microplate reader (Infinite M1000 Pro, Tecan US, Morrisville, NC) in an end-point mode. All determinations were carried out in triplicate.

Section S 1.3 Cell viability assays

The cell lines Hct-116, SW480, MDA-MB-231and MCF-7 were obtained from the Shanghai

Cell Bank at the Chinese Academy of Sciences. Hct-116, SW480, MDA-MB-231 and MCF-7 cell lines were grown on 96-well microtiter plates at a density of 10×10^5 cells/well in DMEM with 10% FBS. DMEM and FBS were obtained from Gibco-Thermo (BRL Co. Ltd., USA). The plates were incubated at 37 °C in a humidified atmosphere of 5% CO_2/95% air overnight. The cells were then exposed to different concentrations of target compounds, 1-MT, epacadostat and incubated for another 48 h. The cells were stained with 10 μl of MTT at incubator for approximately 4 h. The medium was discarded and replaced by 100 mL DMSO. The O.D. value was read at 570/630 nm with a spectrophotometer.

Section S 1.4 Molecular docking

Molecular docking was carried out in Sybyl-X 2.1 on a Windows workstation. The crystal structure of IDO1 protein with inhibitor was retrieved from the RCSB Protein Data Bank (IDO1: 4PK5, HDAC6: 6THV) [15, 35]. Firstly, the 3D structure of the selected compound was built with Sybyl-X 2.1 sketch followed by energy minimization with the MMFF94 force field and Gasteiger-Marsili charges. The Powell's method was used to optimize the geometry with a distance dependent dielectric constant and a termination energy gradient of 0.005 kcal/mol. After extracting the natural ligand, the water molecules were removed from the crystal structure. Subsequently, the protein was prepared with the Biopolymer module implemented in Sybyl. The polar hydrogen atoms were added, and other parameters were established by default to estimate the binding affinity characterized by the Surflex-Dock scores in the software. Before the docking process, the docking model was confirmed with epacadostat docked into the designated target using the default glide settings. Then, the selected compounds were automatically docked into the binding pocket of IDO1 and HDAC6 through an empirical scoring function and a patented search engine in the Surflex docking program. The Surflex-Dock total scores, which were expressed in -log10 (K_d) units to represent binding affinities, were applied to estimate the ligand-receptor interactions of newly designed molecules.

Section S 1.5. Cell cycle measurement

HCT-116 Cells were plated in 6-well plates and allowed to adhere overnight. After incubation overnight, the tumor cells were treated with 5, 10 or 20 μM of compound **10e** and cultured in fresh cell medium for 24 h. Following treatments, the cells were harvested, washed with PBS and fixed

with 75% cold ethanol at 4 °C overnight. After that, fixed cells were washed with PBS. Then, cells were isolated by centrifugation and stained with propidium iodide (KeyGEN BioTECH, China) for 30 min. The samples were measured using flow cytometry and analyzed by Flowjo software.

Section S 1.6 Western blot assay

Total cell lysates from cultured HCT-116 cells after treatment with compound **10e** as mentioned earlier were obtained by lysing the cells in ice-cold RIPA buffer with protease and phosphatase inhibitor and stored at -20°C for future use. The protein concentrations were quantified by the Bradford method (Bio-Rad) using a Multimode Varioscan instrument (Thermo Fischer Scientific). Equal amounts of protein per lane were applied to 12% SDS polyacrylamide gel for electrophoresis and transferred to a polyvinylidene difluoride (PVDF) membrane (Amersham Biosciences). After the membrane was blocked at room temperature for 2 h in blocking solution, primary antibody was added and incubated at 4°C overnight. Ac-α-tubulin, Ac-histone H3 and GAPDH antibodies were purchased from Imgenex, USA. After three TBST washes, the membrane was incubated with corresponding horseradish peroxidase-labeled secondary antibody (1:2,000) (Santa Cruz) at room temperature for 1 h. Membranes were washed with TBST three times for 15 min and the protein bands were detected with chemiluminescence reagent (Thermo Fischer Scientific). The X-ray films were developed using developer and fixed with fixer solution.

Section S 1.7 In vivo antitumor efficacy

The *in vivo* antitumor activity of compound **10e** was evaluated in the mouse colon carcinoma cell line CT-26 in BALB/c mice as described previously [43, 44]. Five-week-old male BALB/c mice were purchased from the Shanghai Ling Chang biotechnology company (China), and tumors were induced through subcutaneous injection of 1×10^7 cells in 100 μL of sterile PBS into the dorsal region. The animals were divided into four groups, starting on the second day. When the tumors reached a volume of 50−100 mm^3 in all mice, the first group was administered intraperitoneally with an equivalent volume of 5% dextrose as the vehicle control. The second group was treated with SAHA at a dose of 150 mg/kg body weight once every 3 days for 3 weeks. The third group was treated with complex **10e** at doses of 150 mg/kg body weight once every 3 days for 3 weeks, respectively. All compounds were dissolved in vehicle. Tumor volumes and body weights were

recorded every other day after drug treatment. All mice were sacrificed after 3 weeks of treatment, and the tumor volumes were measured with electronic digital calipers and examined by measurement of the length (A) and width (B) to calculate the volume ($V = AB^2/2$).

^{1}H NMR, ^{13}C NMR and HR-MS of compounds**:**

7a

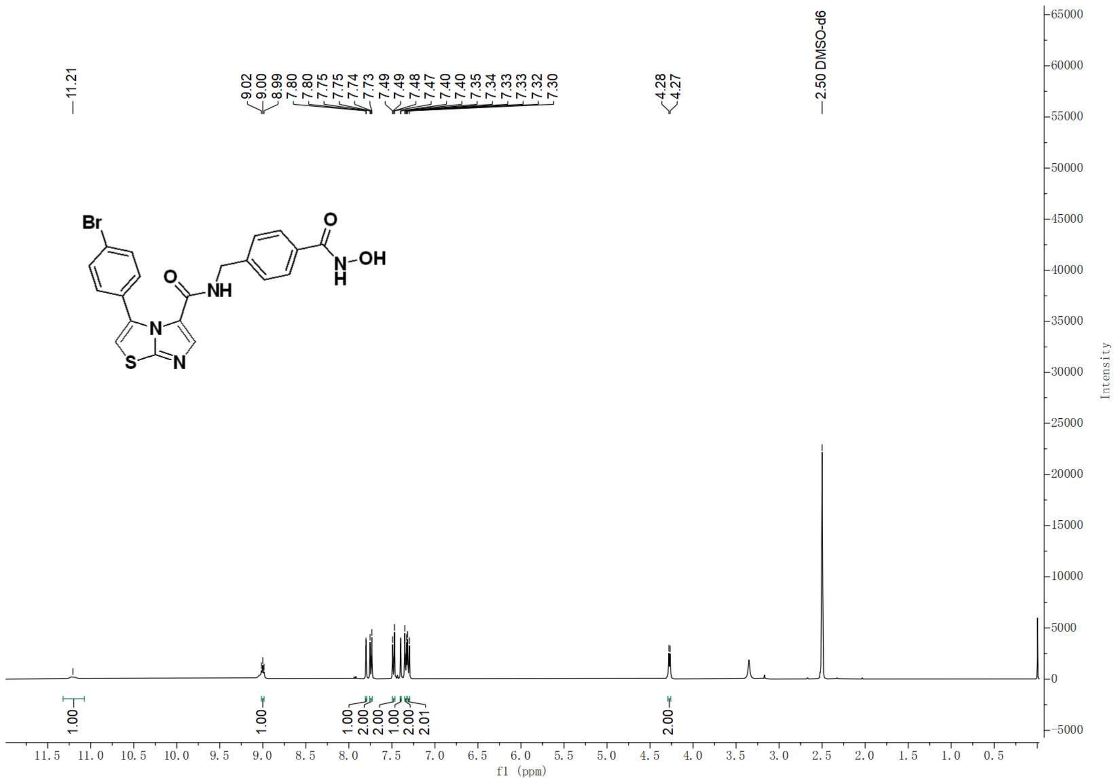

Figure S1. ^{1}H NMR Spectrum of compound **7a**.

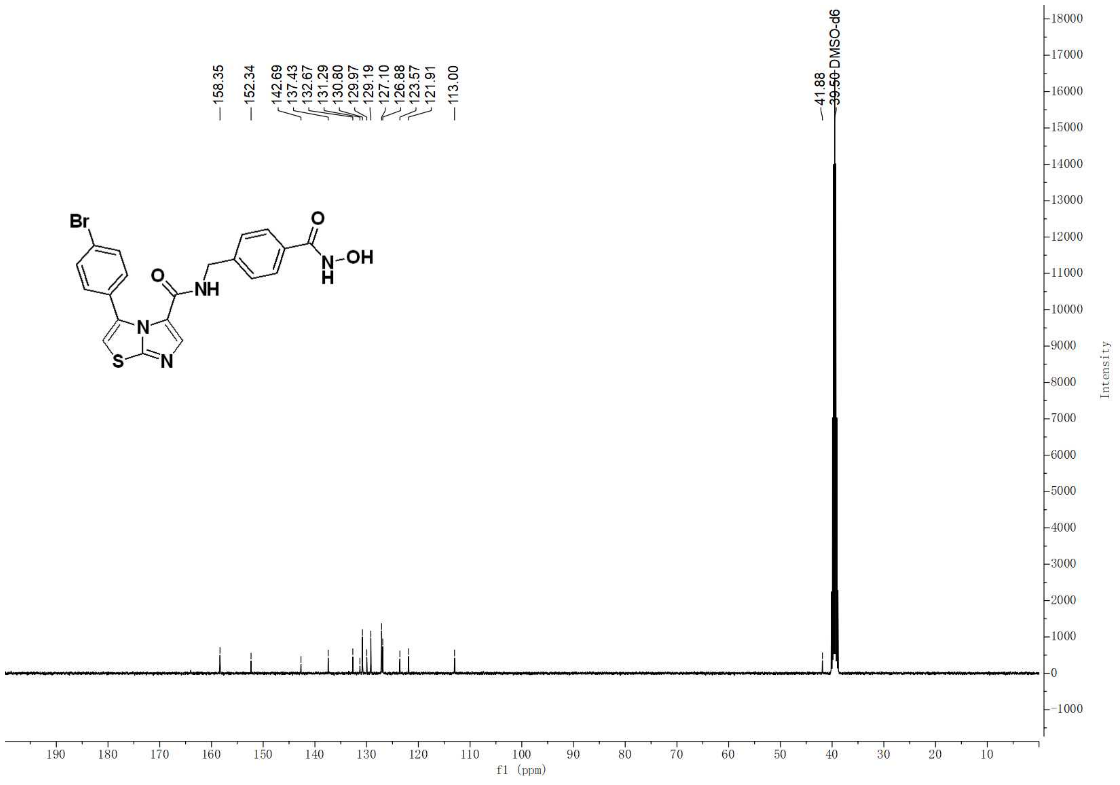

Figure S2. ^{13}C NMR Spectrum of compound **7a**.

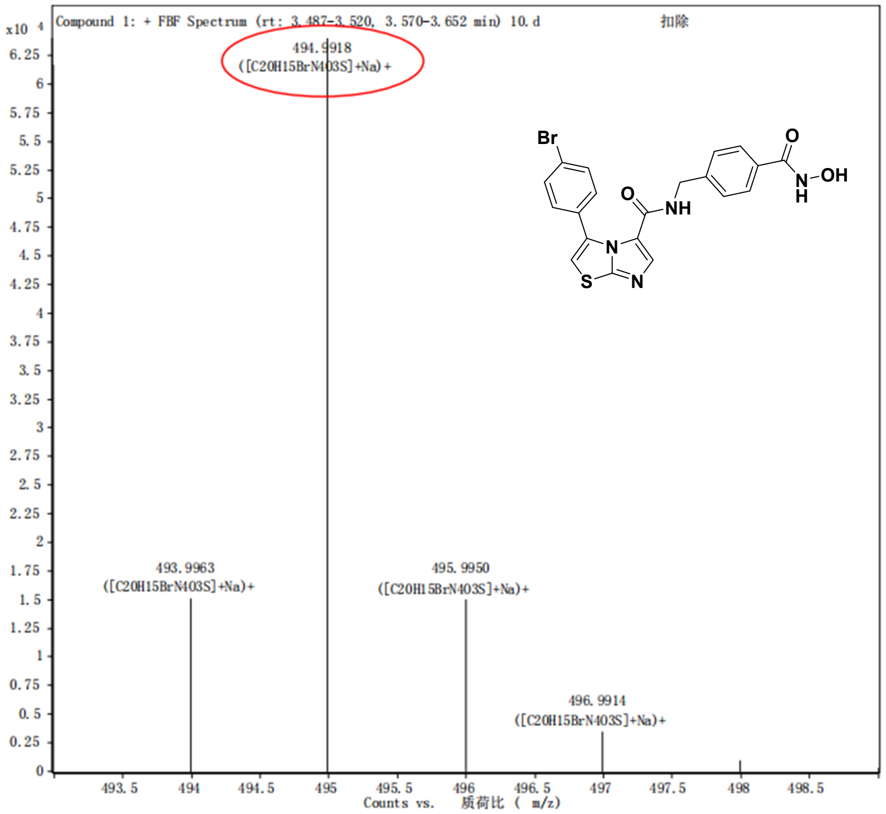

Figure S3. HRMS Spectrum of compound **7a**.

7b

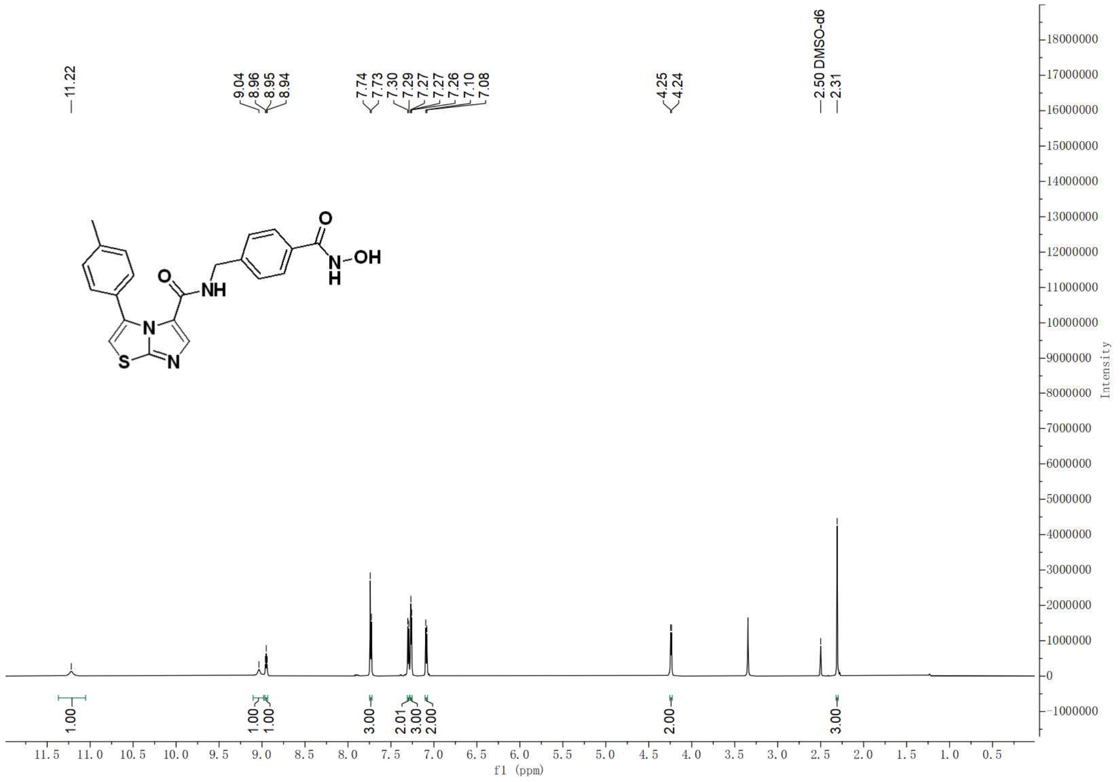

Figure S4. ^{1}H NMR Spectrum of compound **7b**.

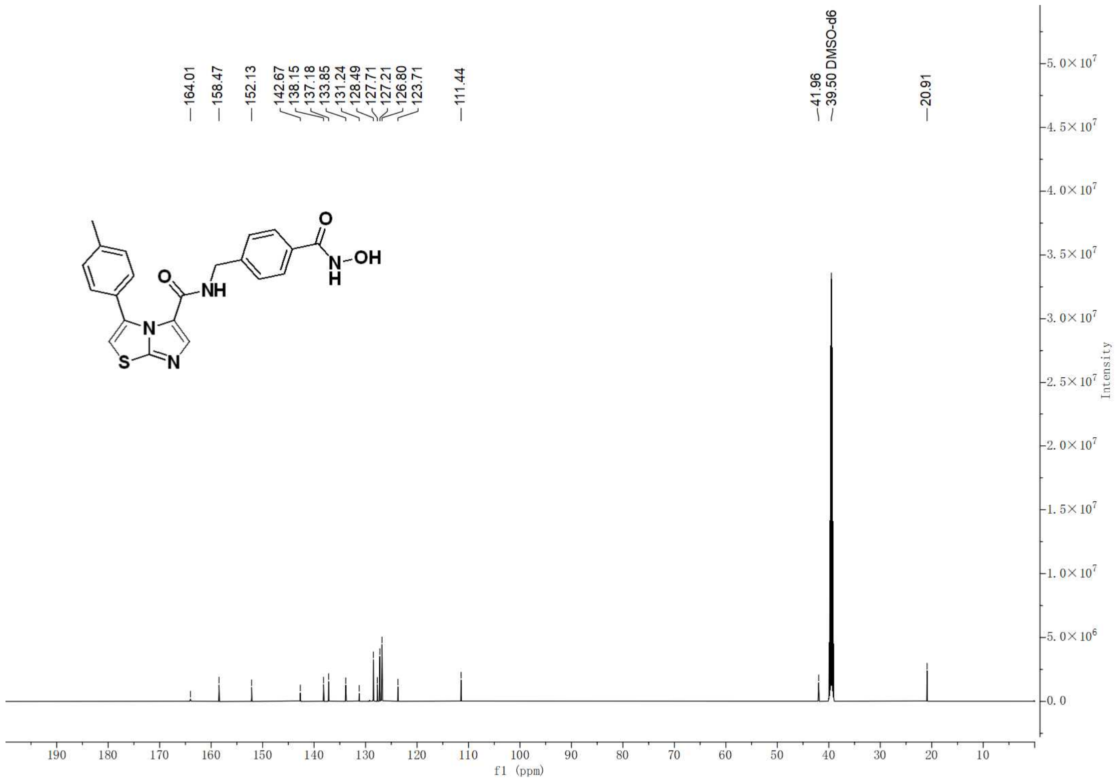

Figure S5. ^{13}C NMR Spectrum of compound **7b**.

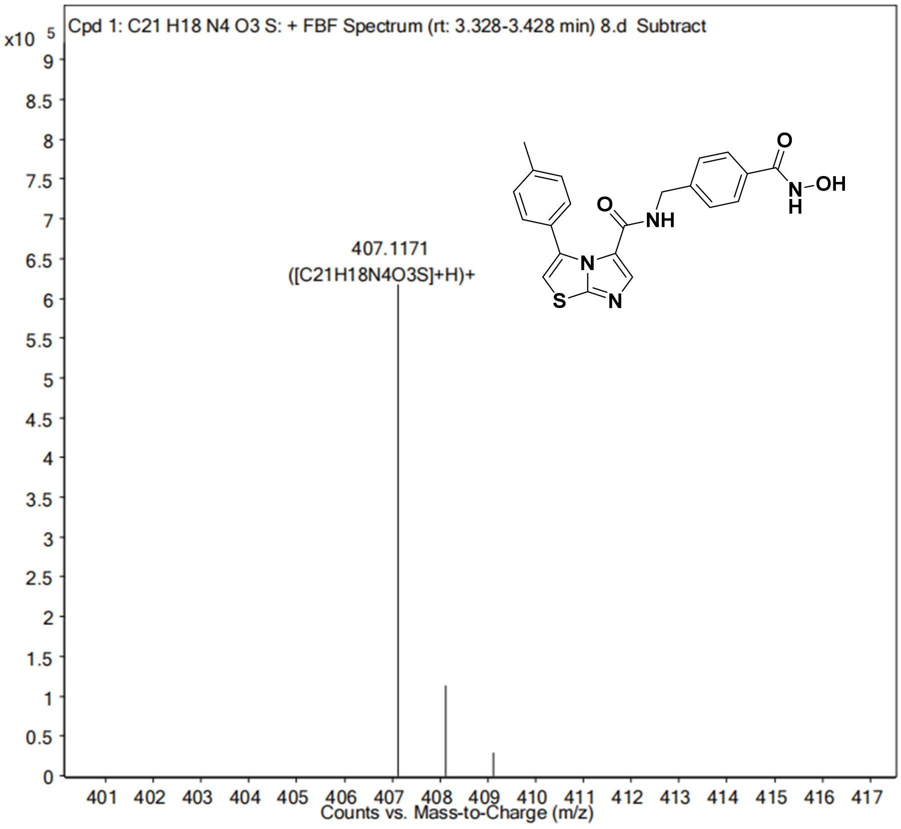

Figure S6. HRMS Spectrum of compound **7b**.

10a

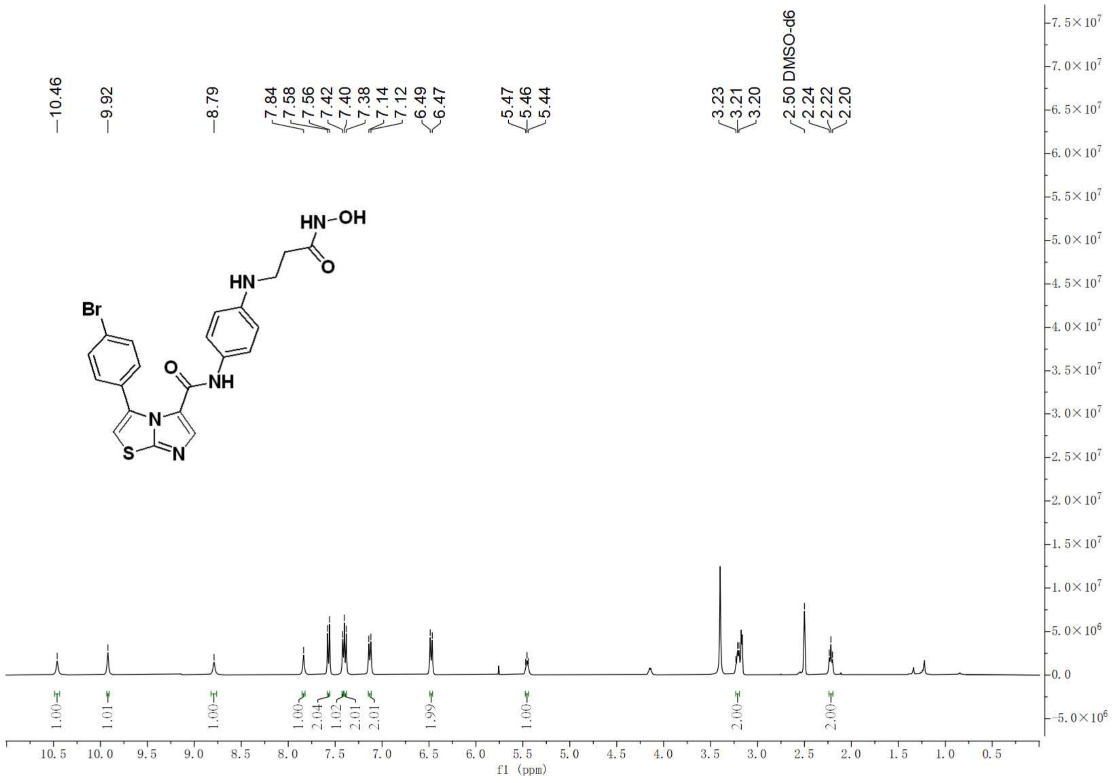

Figure S7. ^{1}H NMR Spectrum of compound **10a**.

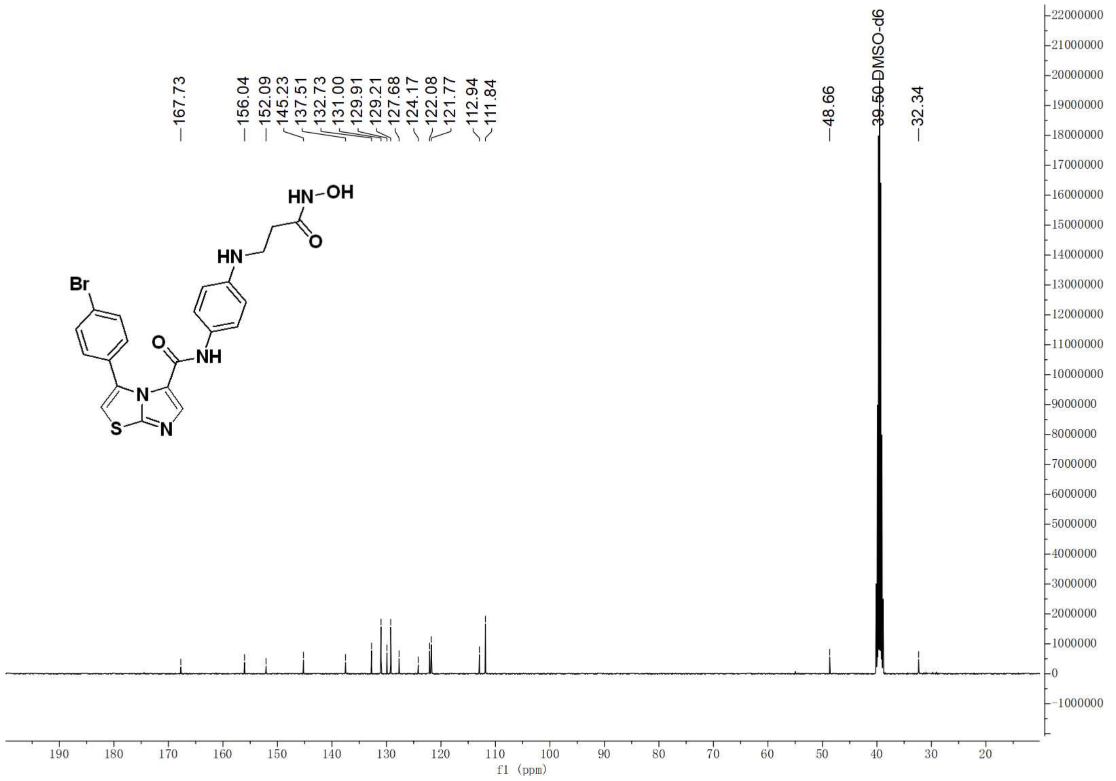

Figure S8. ^{13}C NMR Spectrum of compound **10a**.

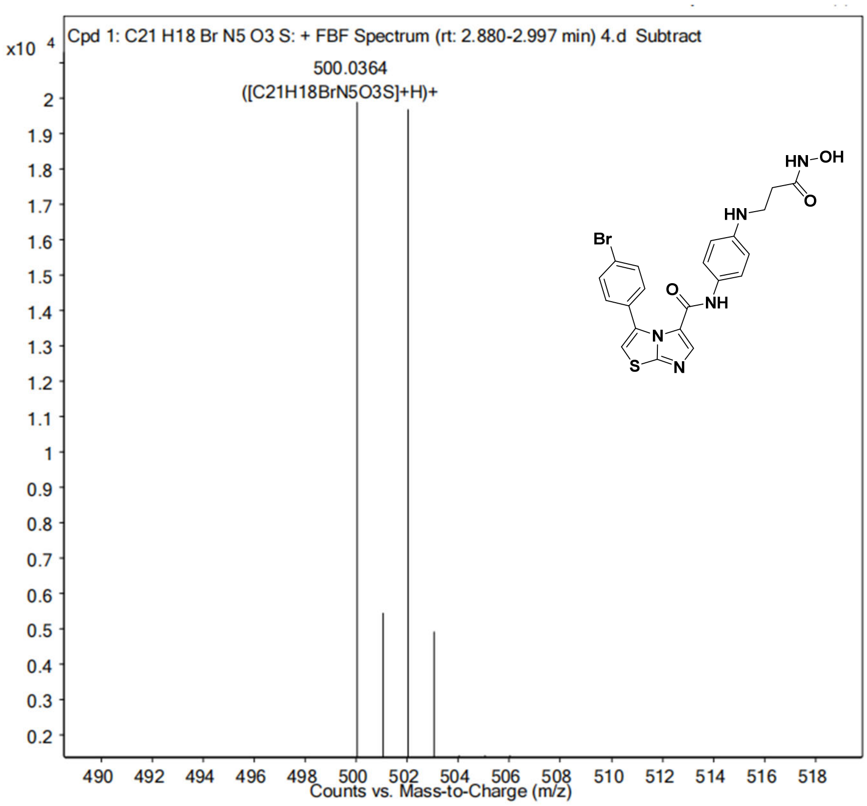

Figure S9. HRMS Spectrum of compound **10a**.

10b

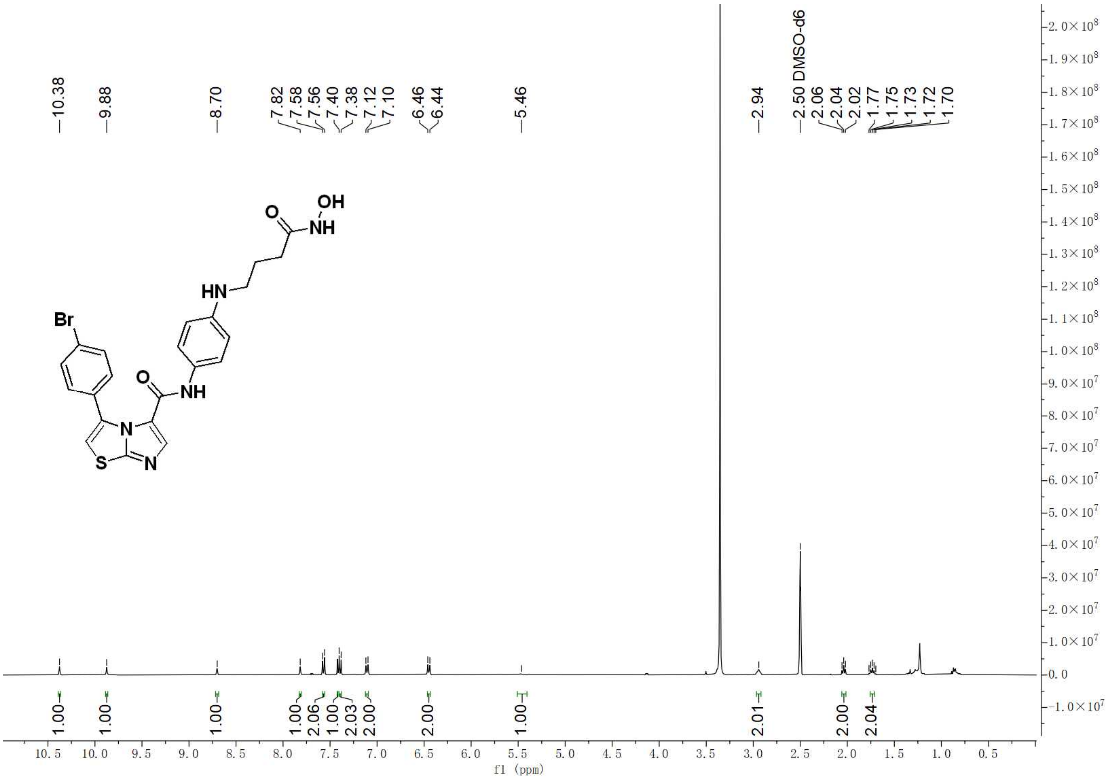

Figure S10. ^{1}H NMR Spectrum of compound **10b**.

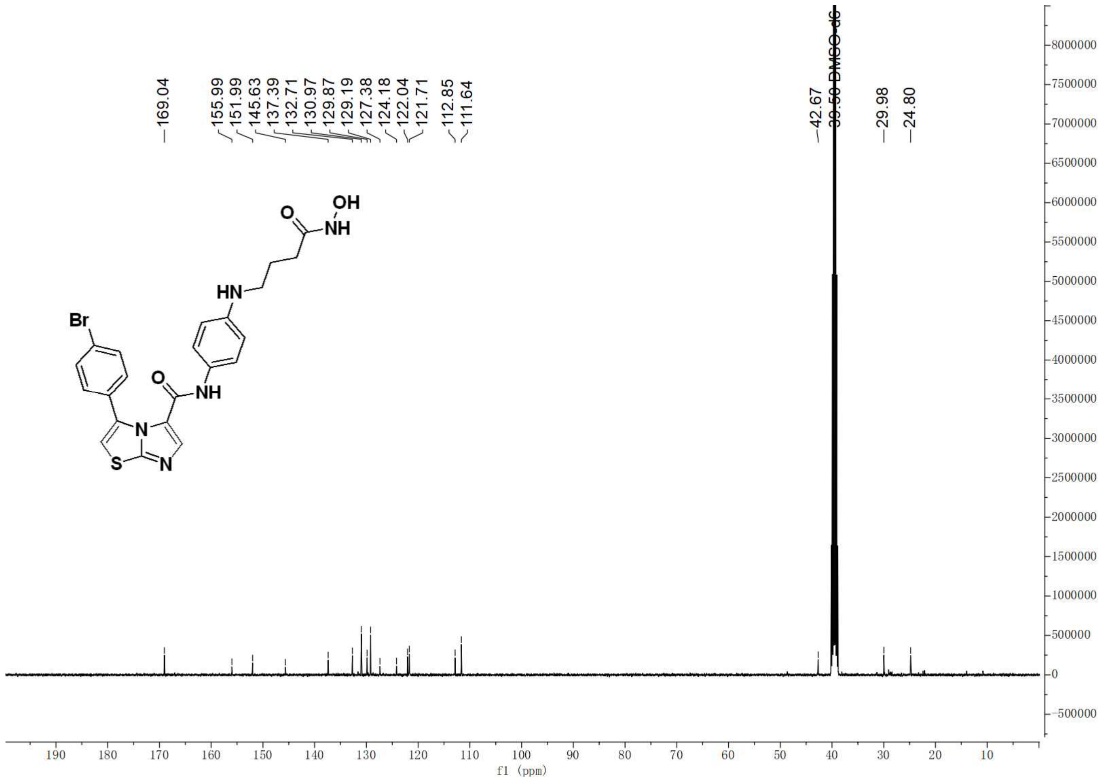

Figure S11. ^{13}C NMR Spectrum of compound **10b**.

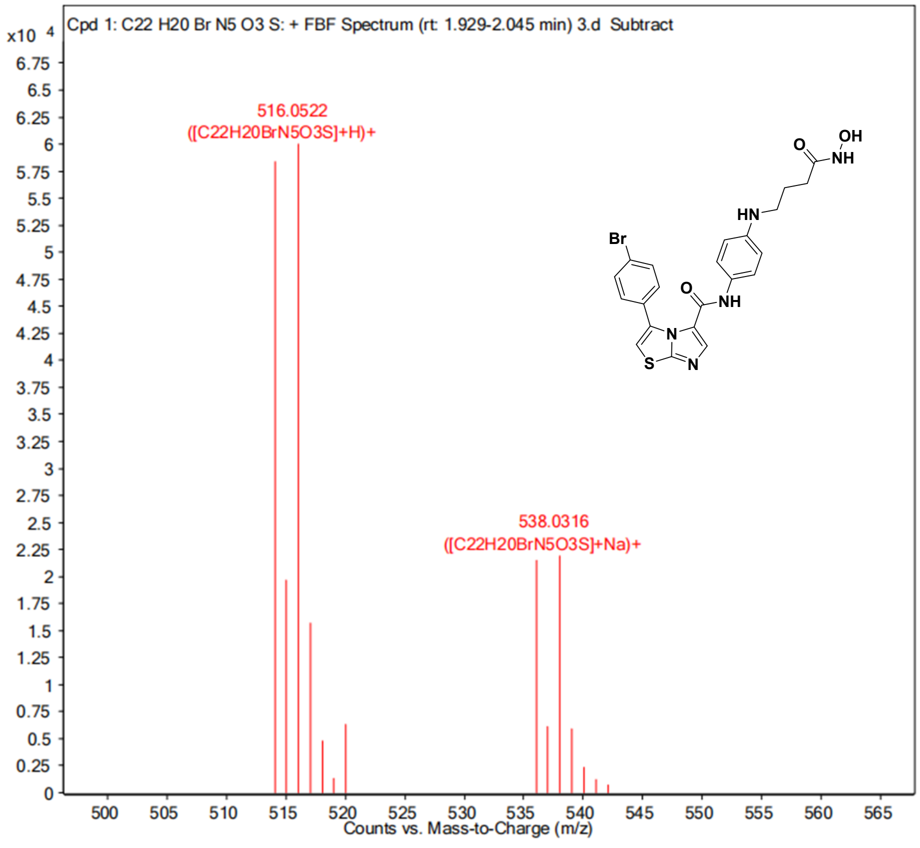

Figure S12. HRMS Spectrum of compound **10b**.

10c

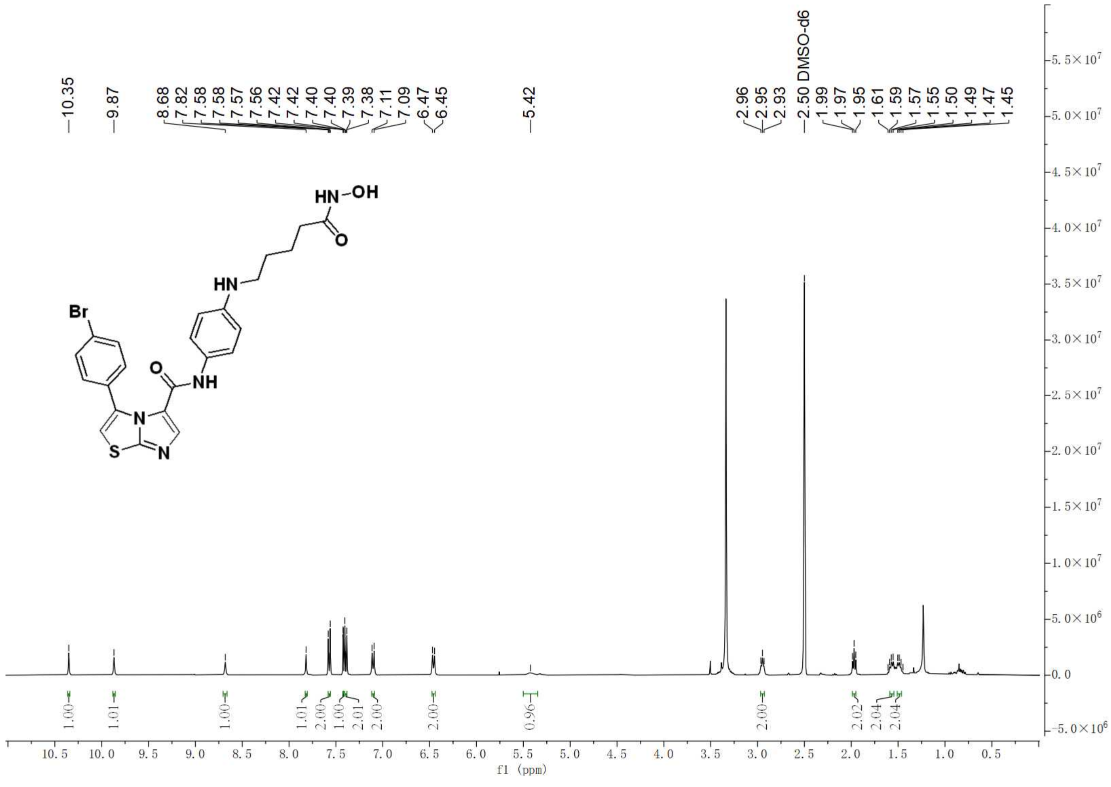

Figure S13. ^{1}H NMR Spectrum of compound **10c**.

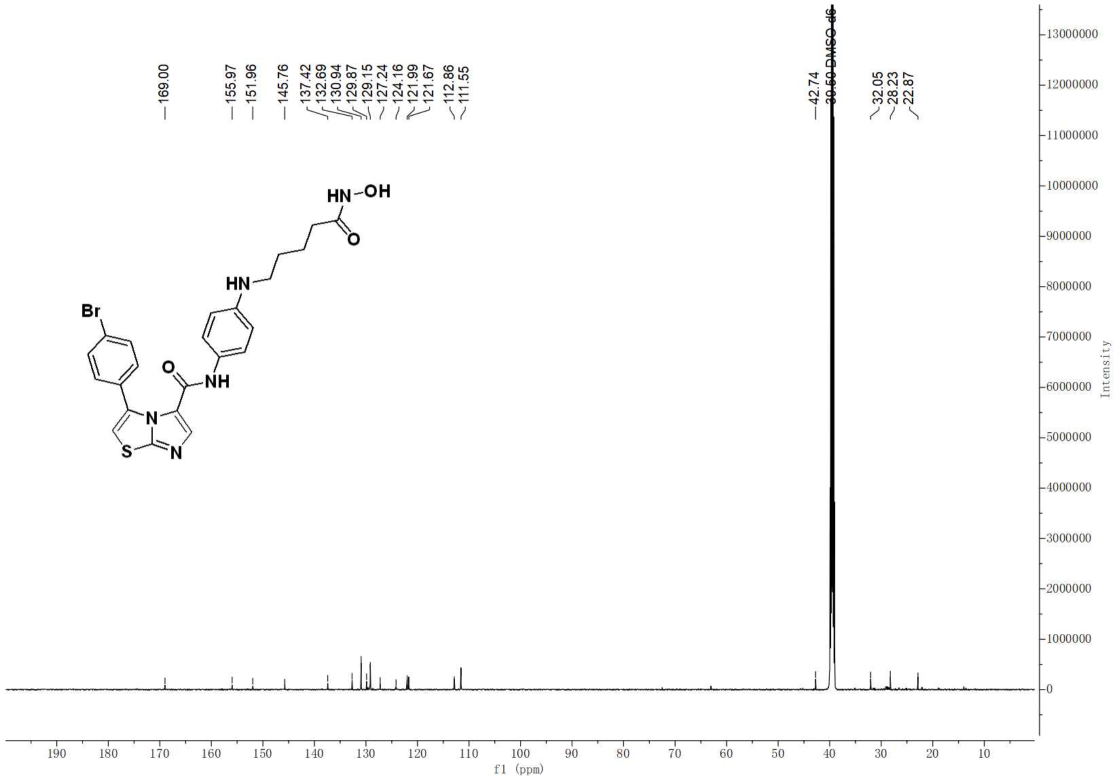

Figure S14. ^{13}C NMR Spectrum of compound **10c**.

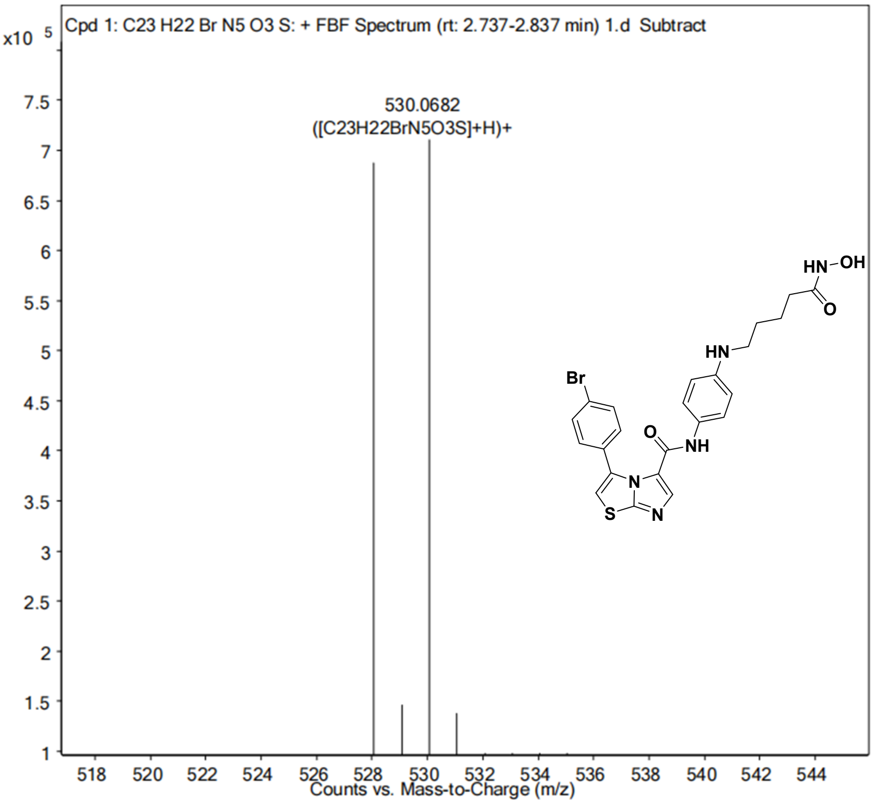

Figure S15. HRMS Spectrum of compound **10c**.

10d

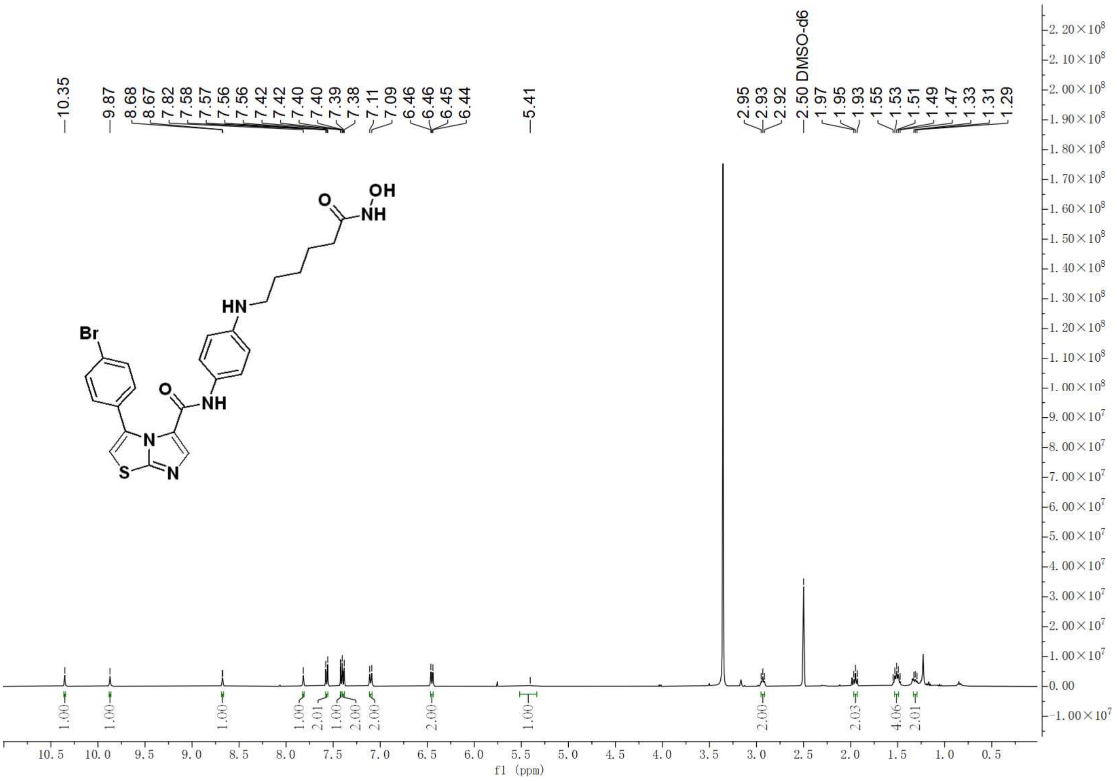

Figure S16. ^{1}H NMR Spectrum of compound **10d**.

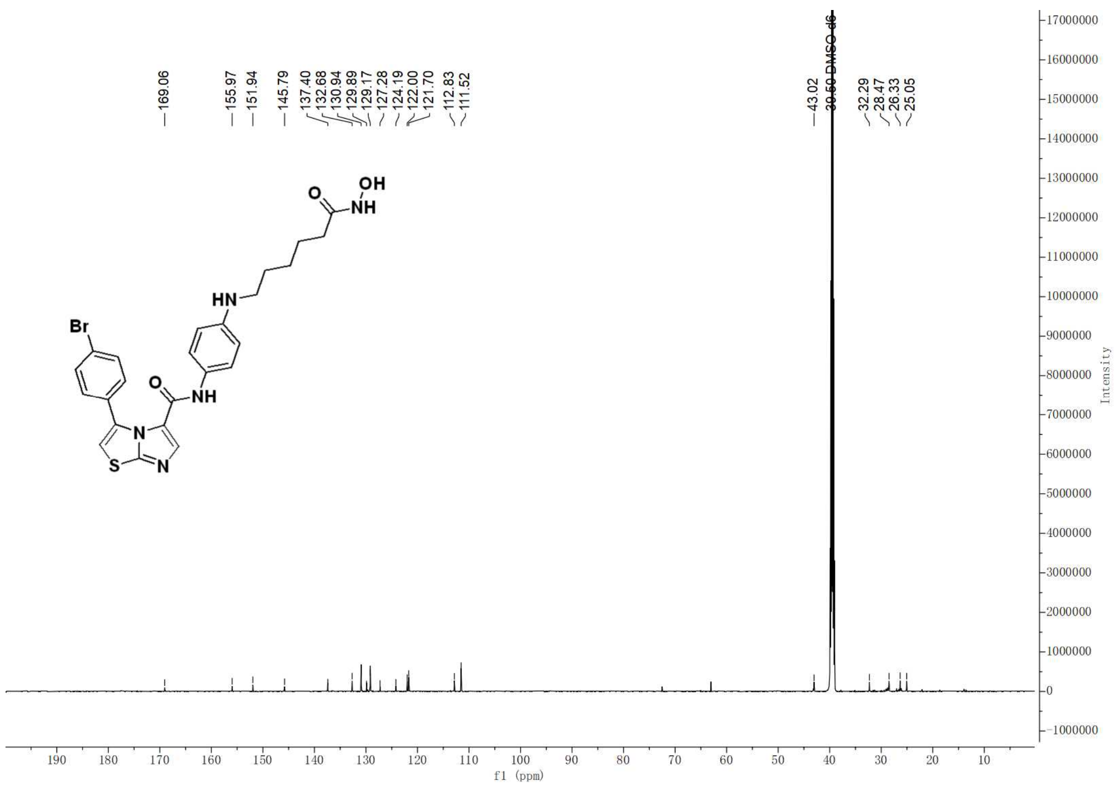

Figure S17. ^{13}C NMR Spectrum of compound **10d**.

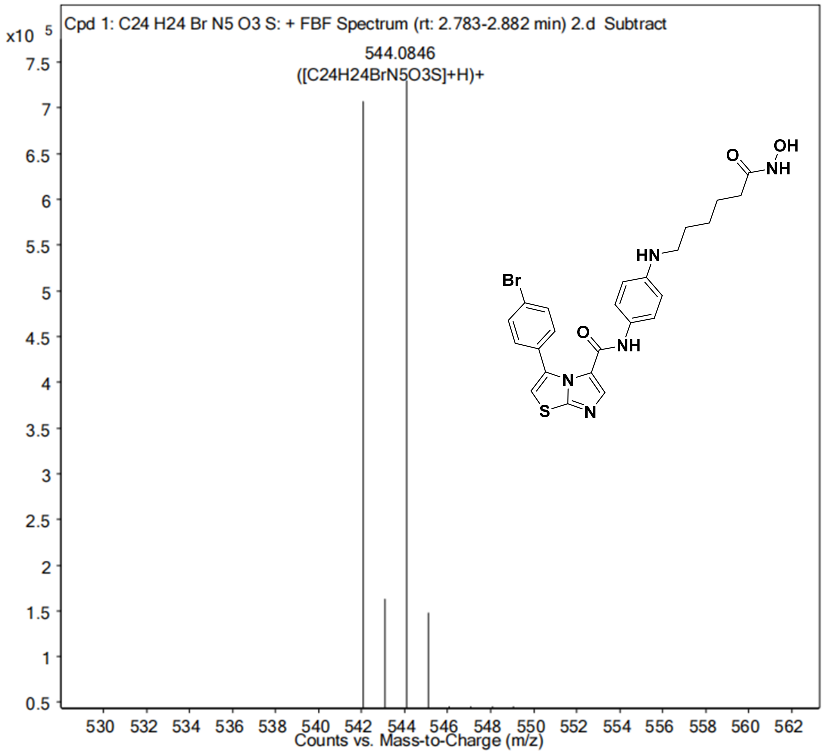

Figure S18. HRMS Spectrum of compound **10d**.

10e

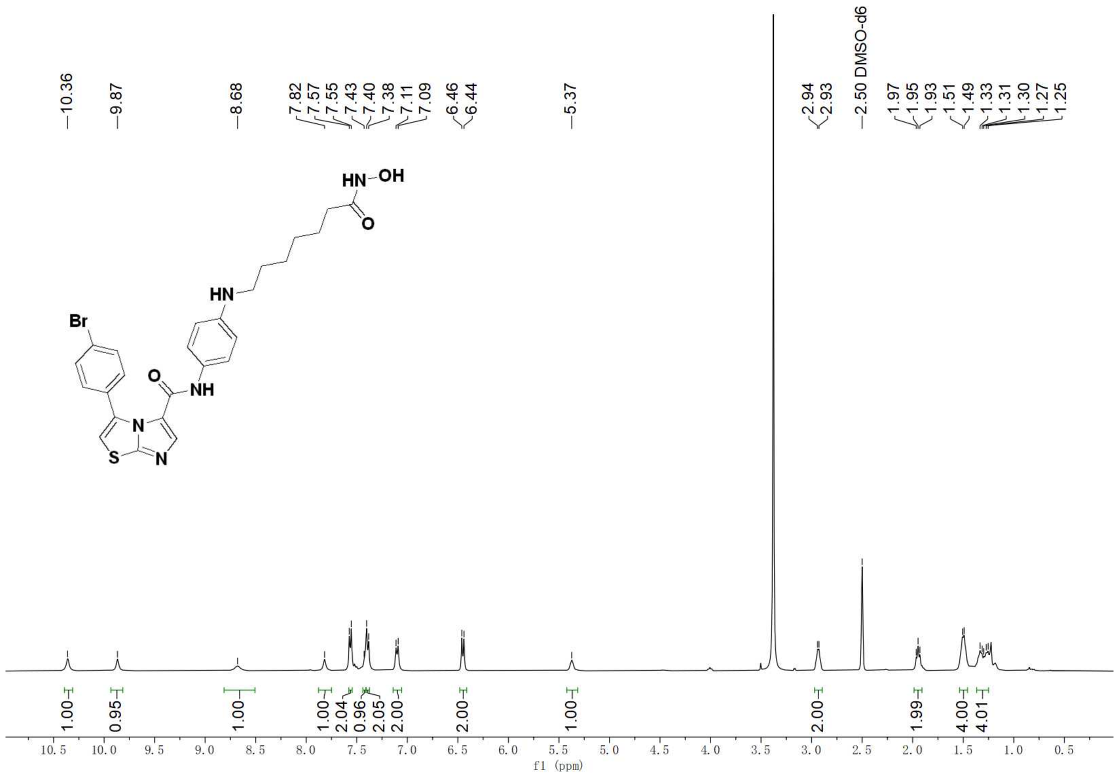

Figure S19. ^{1}H NMR Spectrum of compound **10e**.

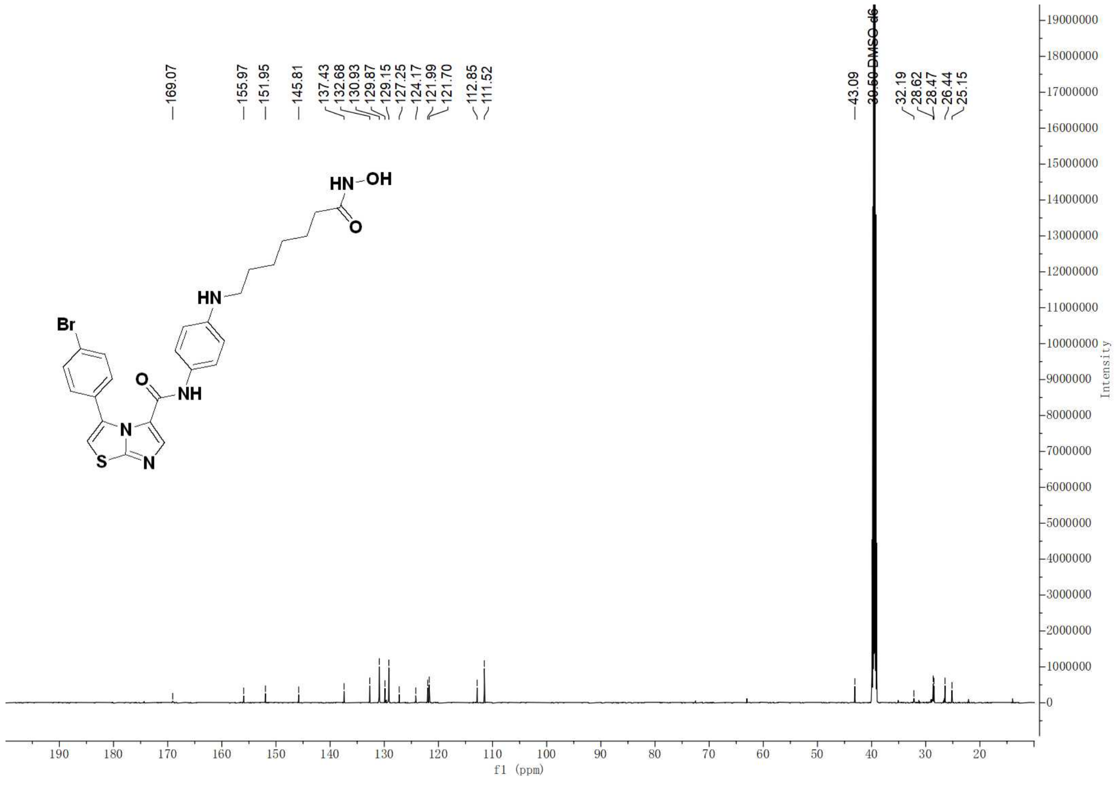

Figure S20. ^{13}C NMR Spectrum of compound **10e**.

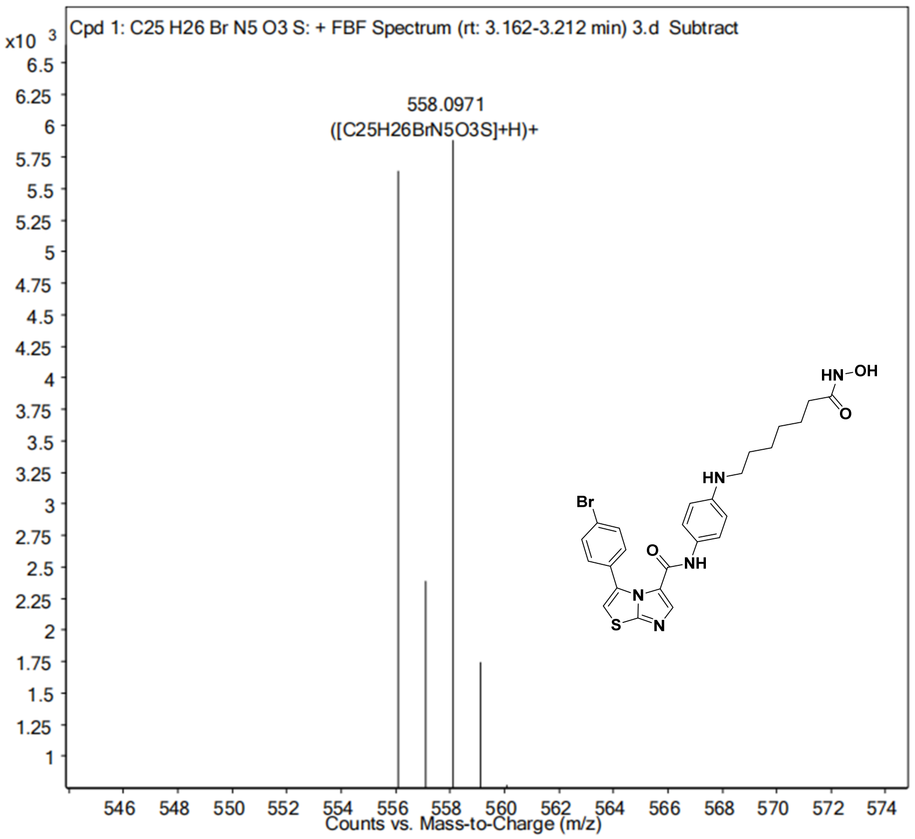

Figure S21. HRMS Spectrum of compound **10e**.

10f

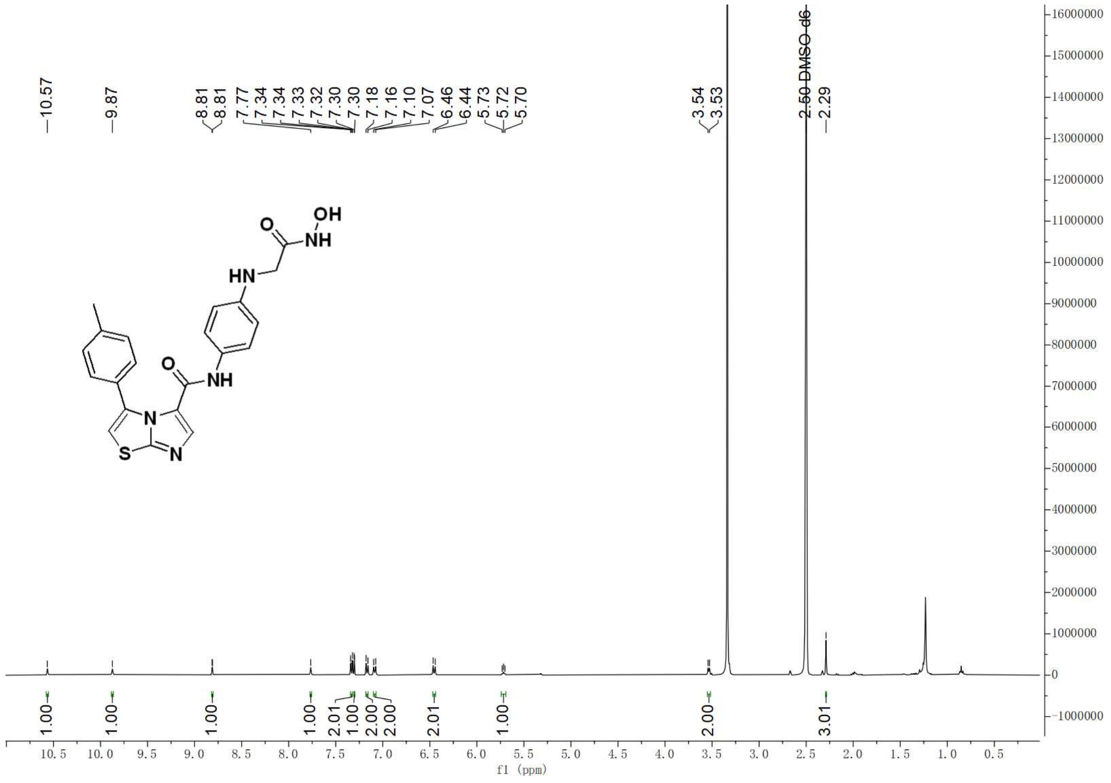

Figure S22. ^{1}H NMR Spectrum of compound **10f**.

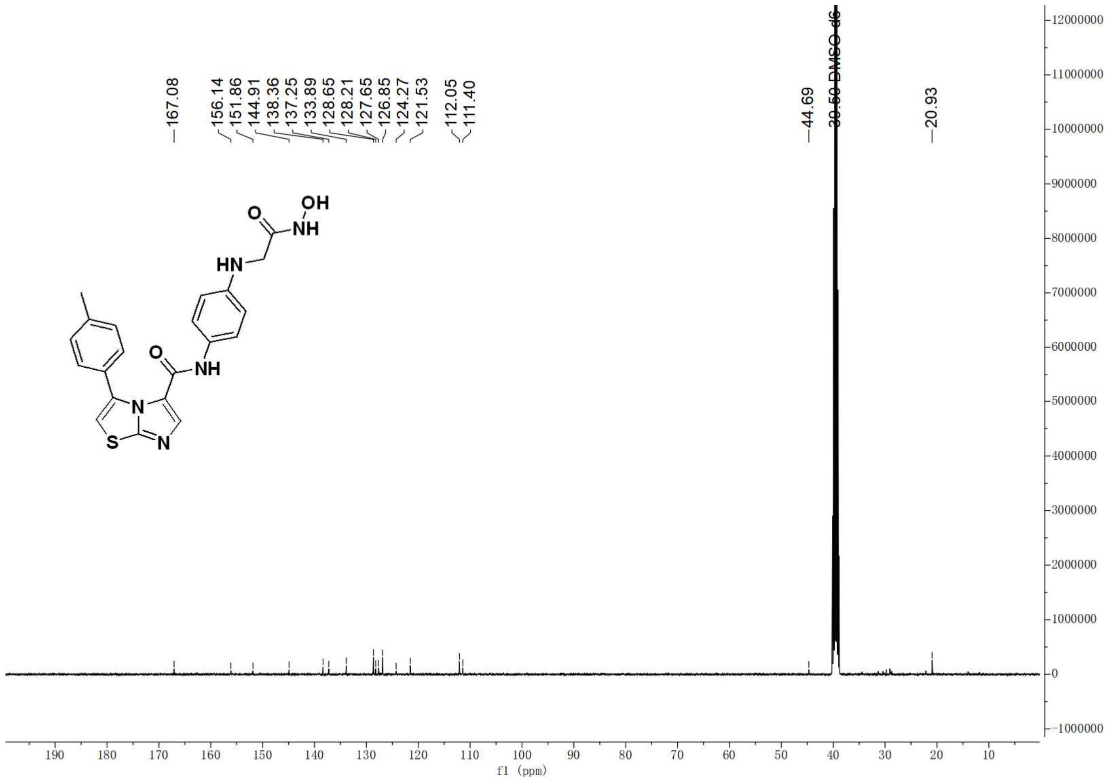

Figure S23. ^{13}C NMR Spectrum of compound **10f**.

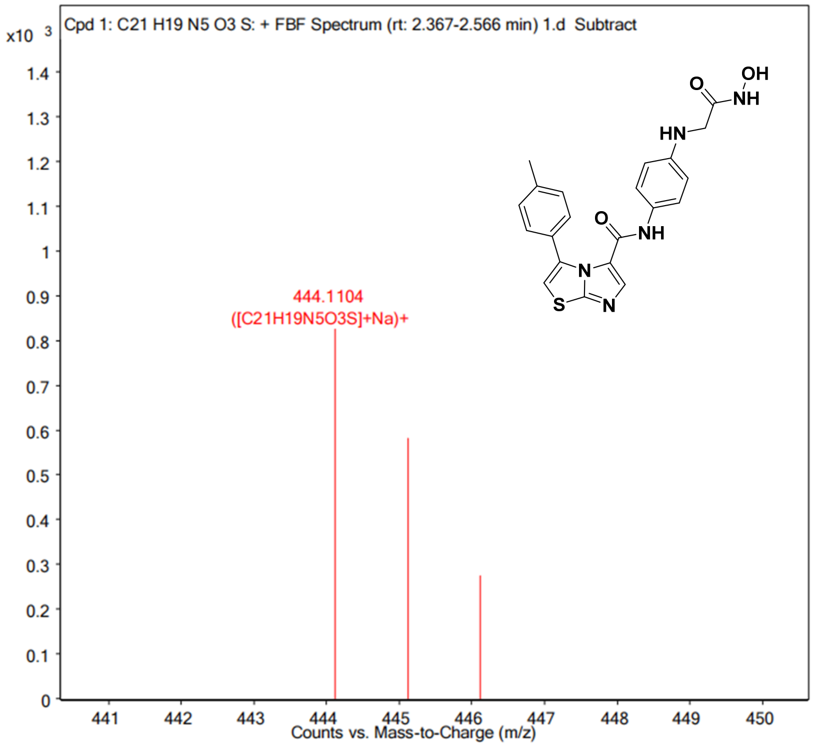

Figure S24. HRMS Spectrum of compound **10f**.

10g

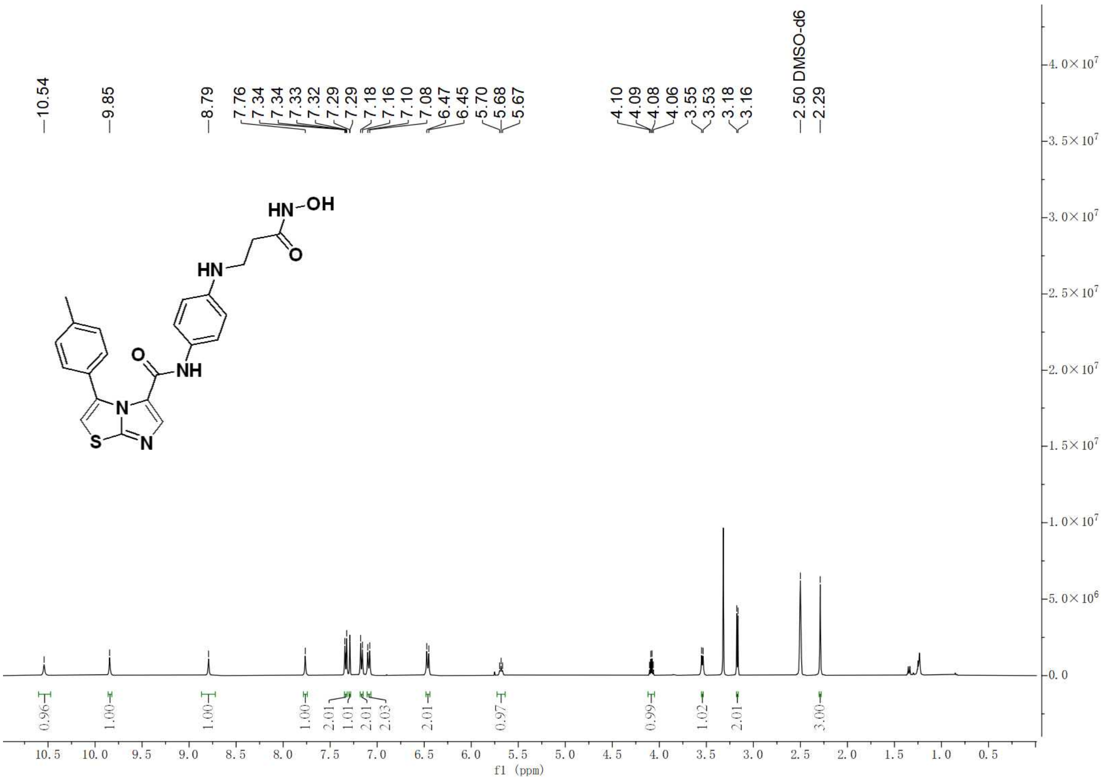

Figure S25. ^{1}H NMR Spectrum of compound **10g**.

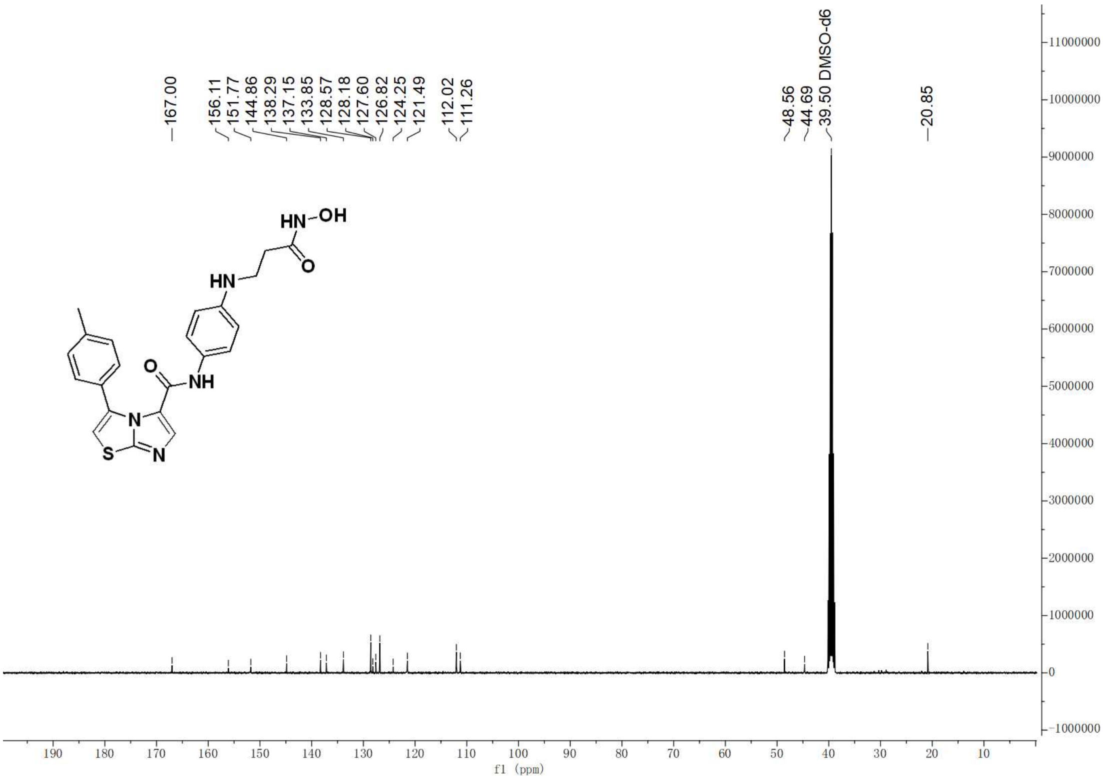

Figure S26. ^{13}C NMR Spectrum of compound **10g**.

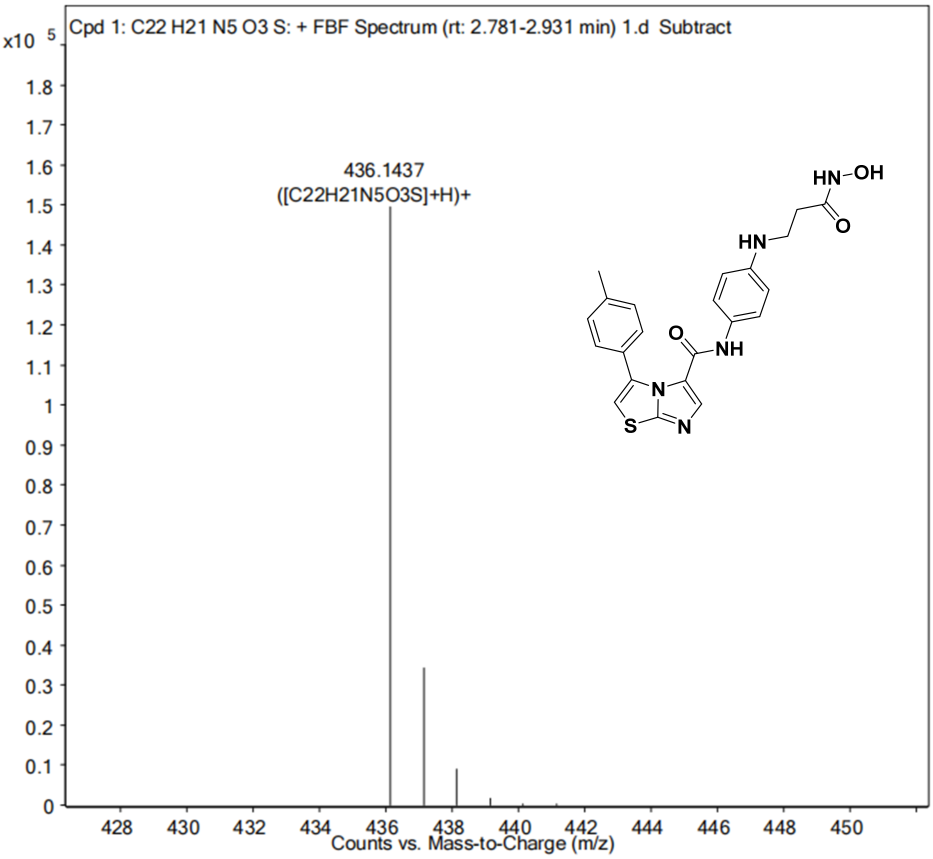

Figure S27. HRMS Spectrum of compound **10g**.

10h

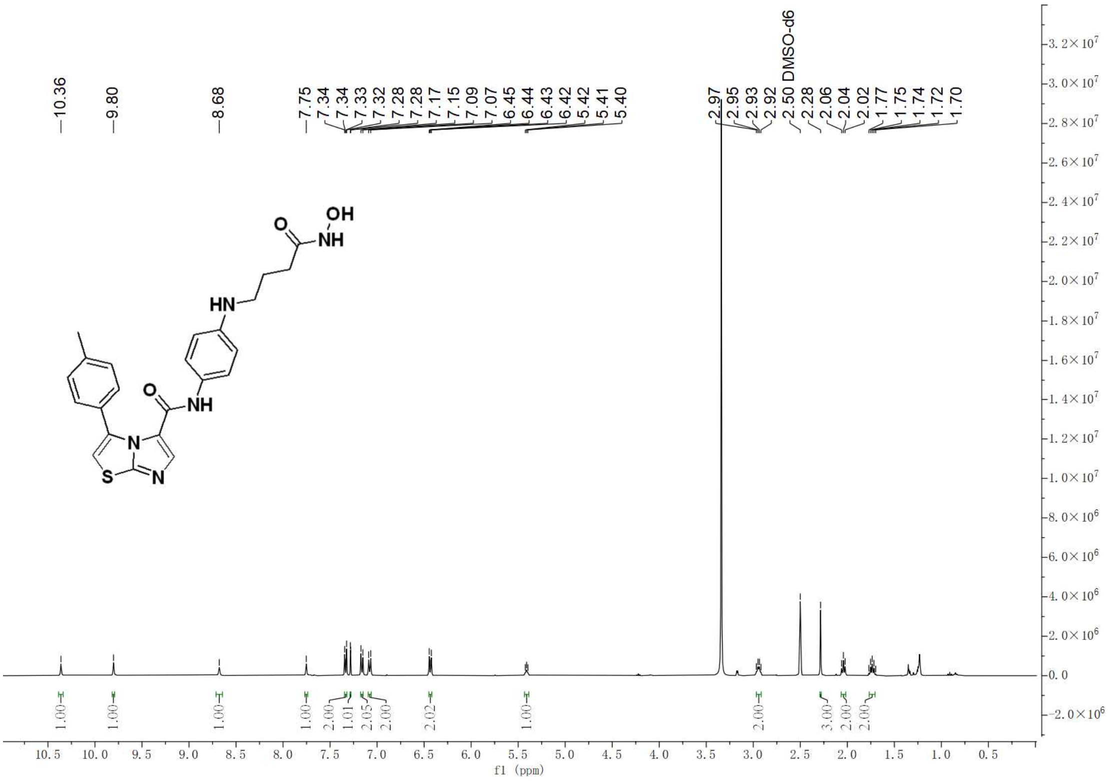

Figure S28. ^{1}H NMR Spectrum of compound **10h**.

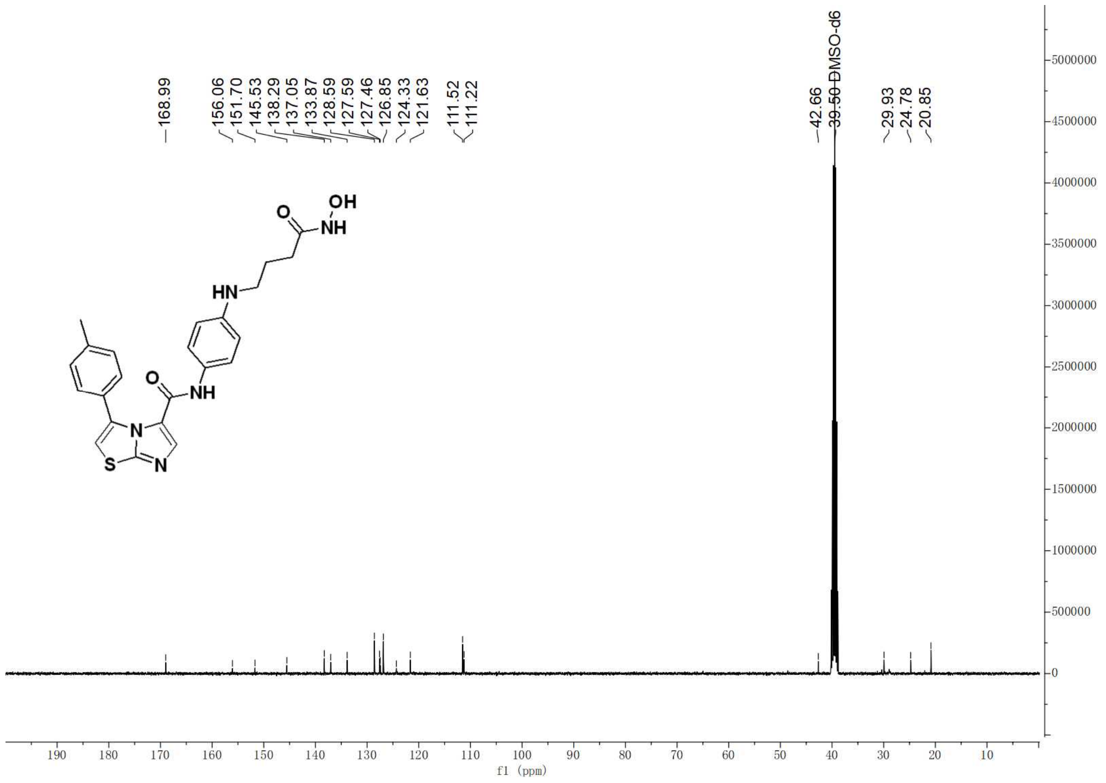

Figure S29. ^{13}C NMR Spectrum of compound **10h**.

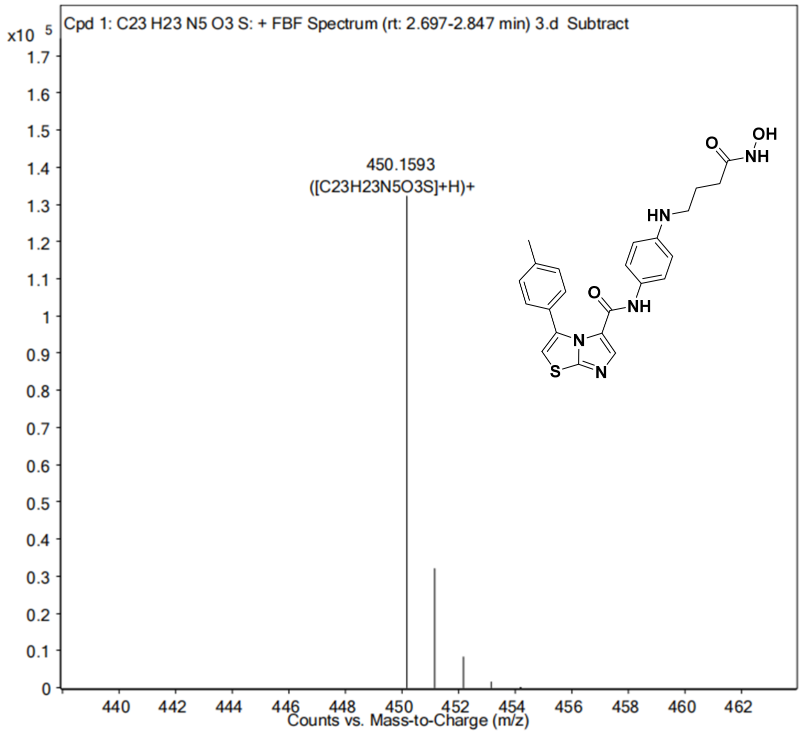

Figure S30. HRMS Spectrum of compound **10h**.

10i

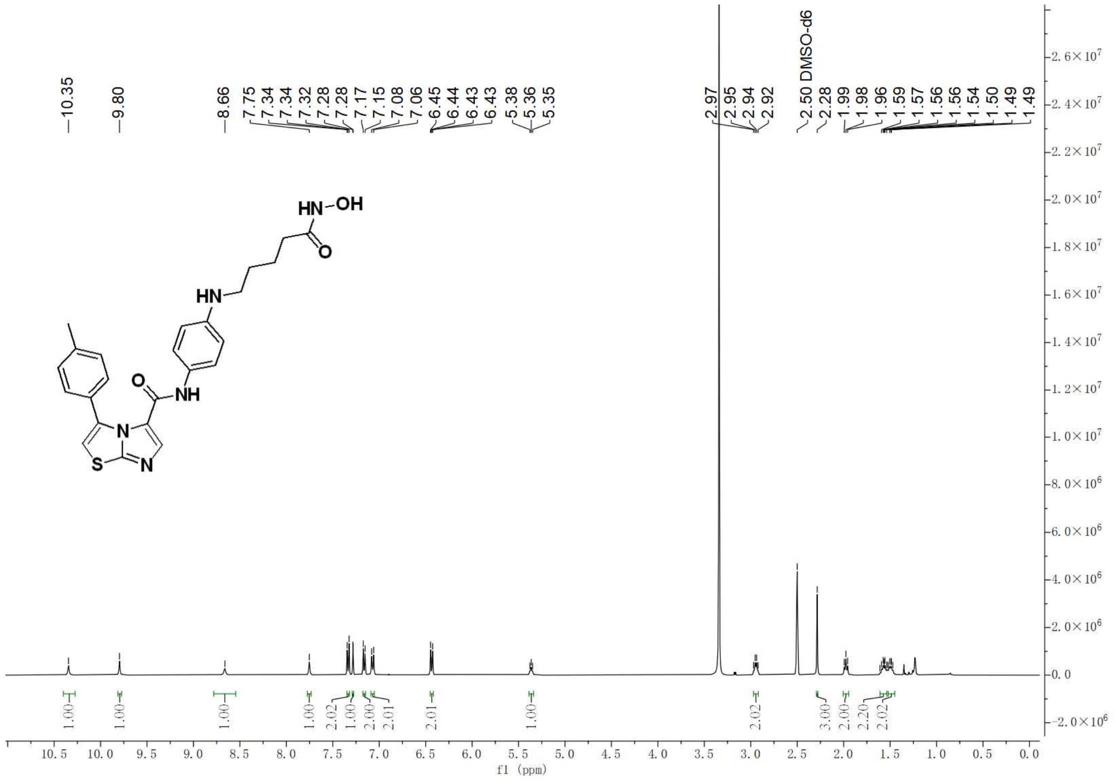

Figure S31. ^{1}H NMR Spectrum of compound **10i**.

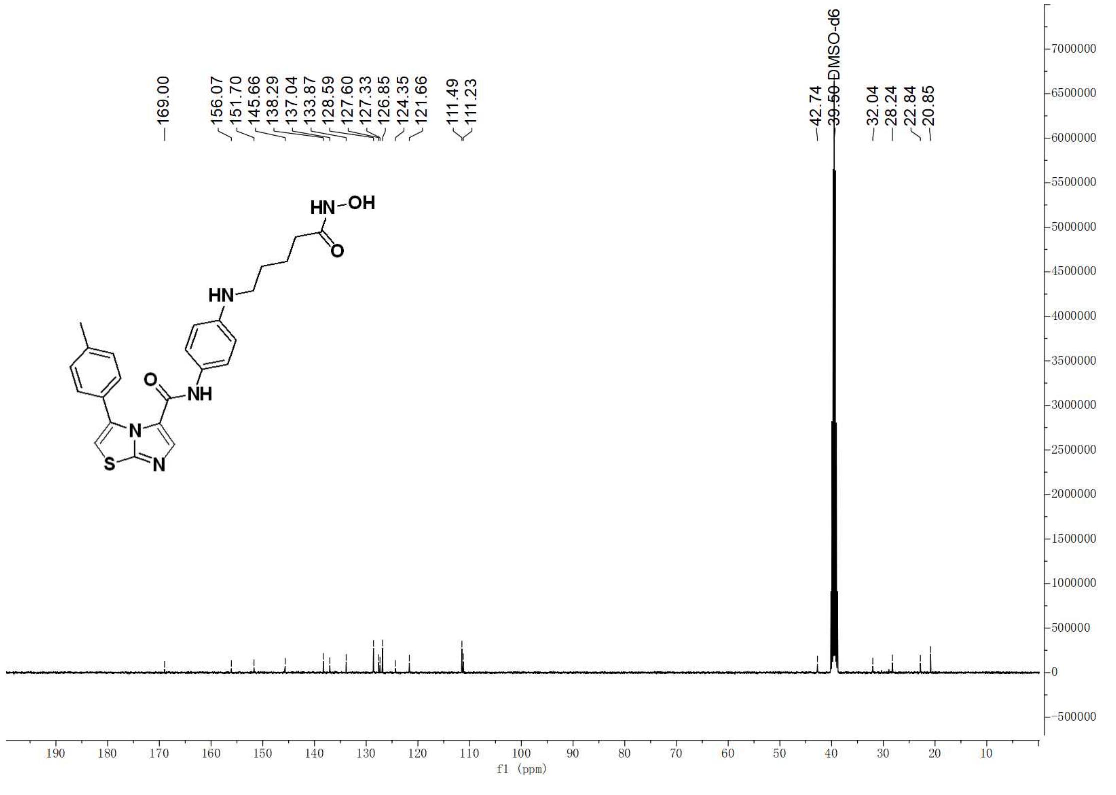

Figure S32. ^{13}C NMR Spectrum of compound **10i**.

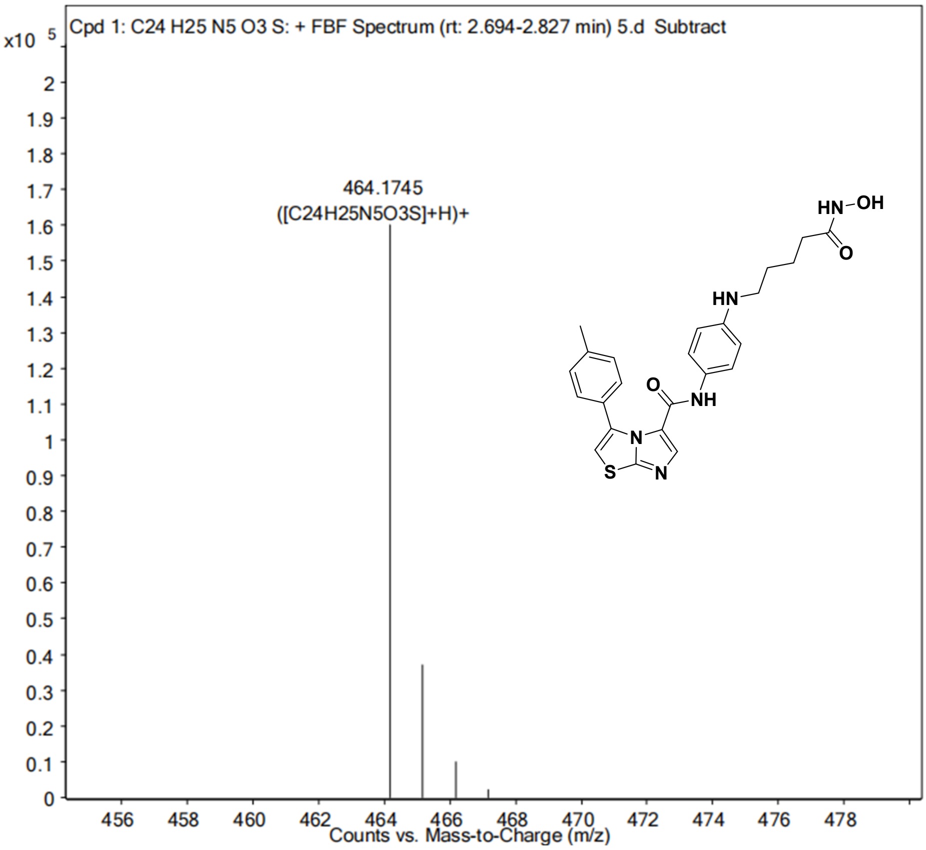

Figure S33. HRMS Spectrum of compound **10i**.

10j

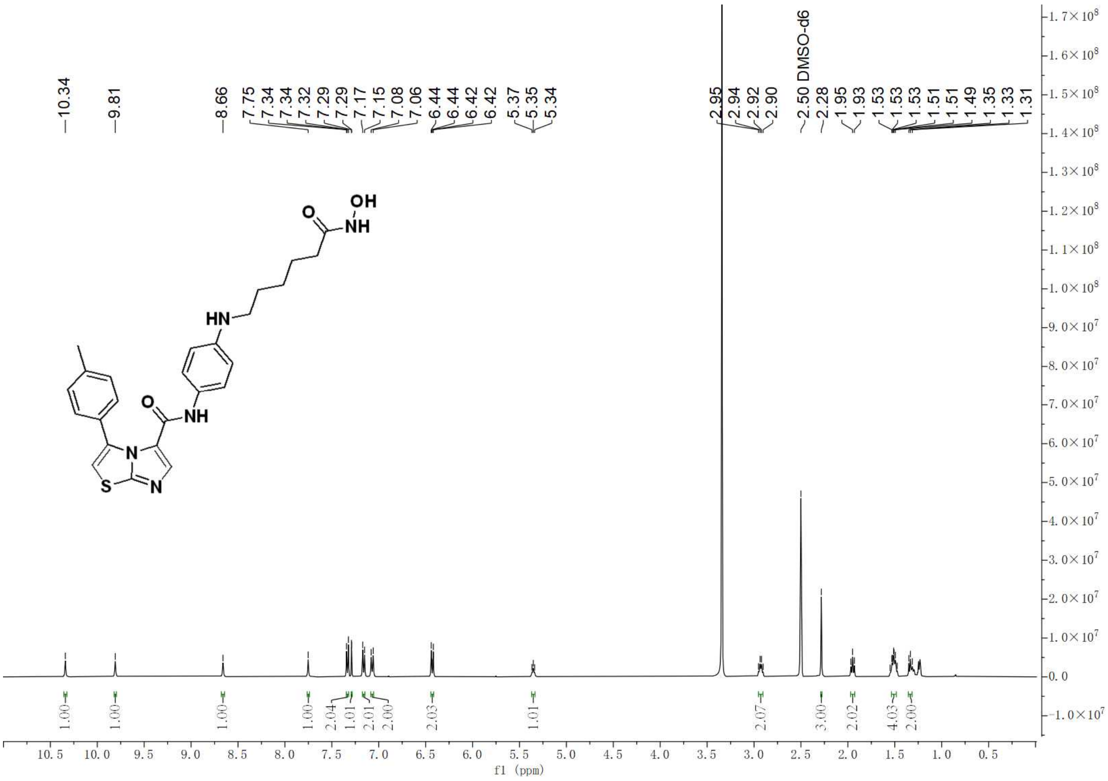

Figure S34. ^{1}H NMR Spectrum of compound **10j**.

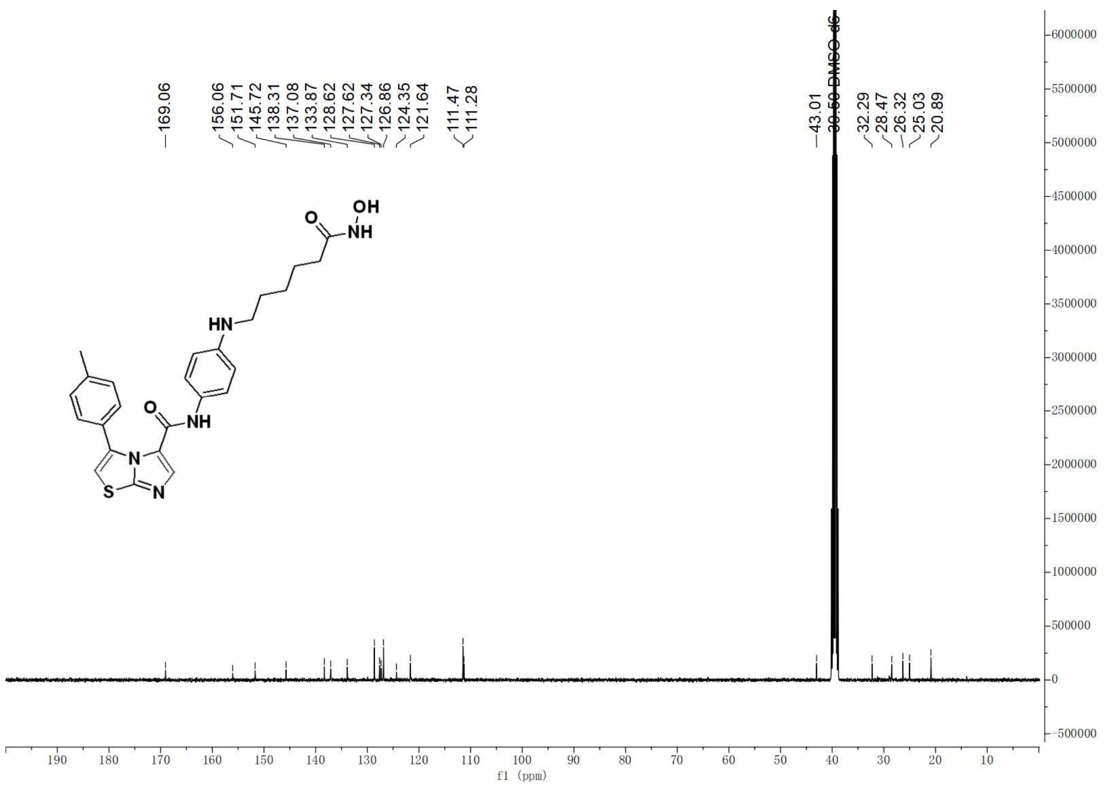

Figure S35. ^{13}C NMR Spectrum of compound **10j**.

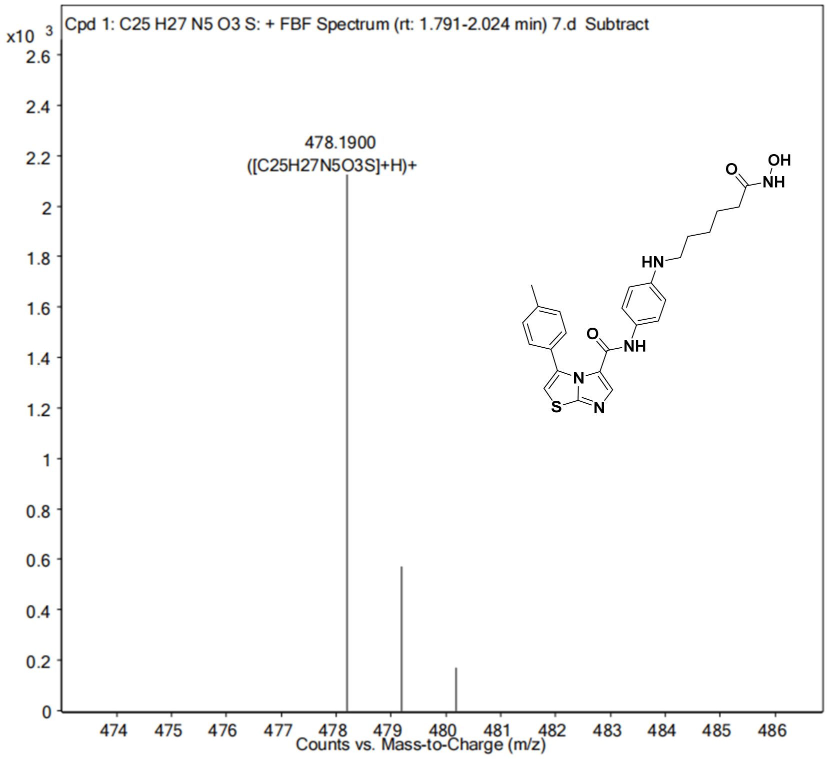

Figure S36. HRMS Spectrum of compound **10j**.

10k

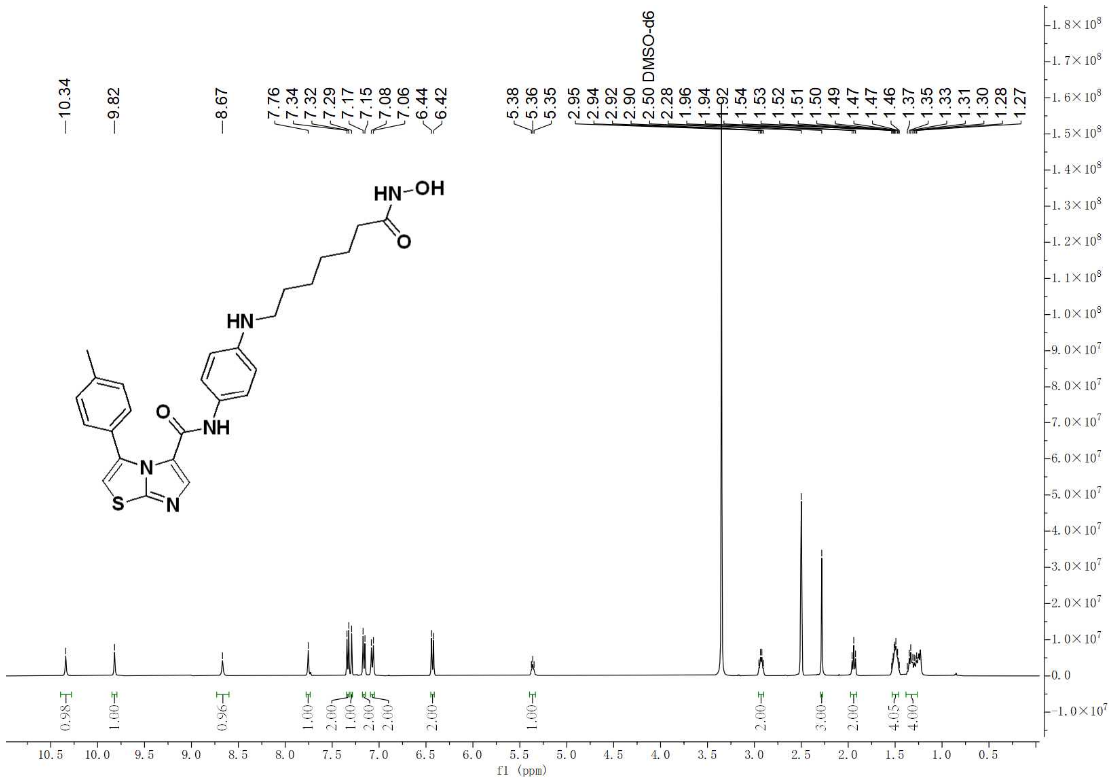

Figure S37. ^{1}H NMR Spectrum of compound **10k**.

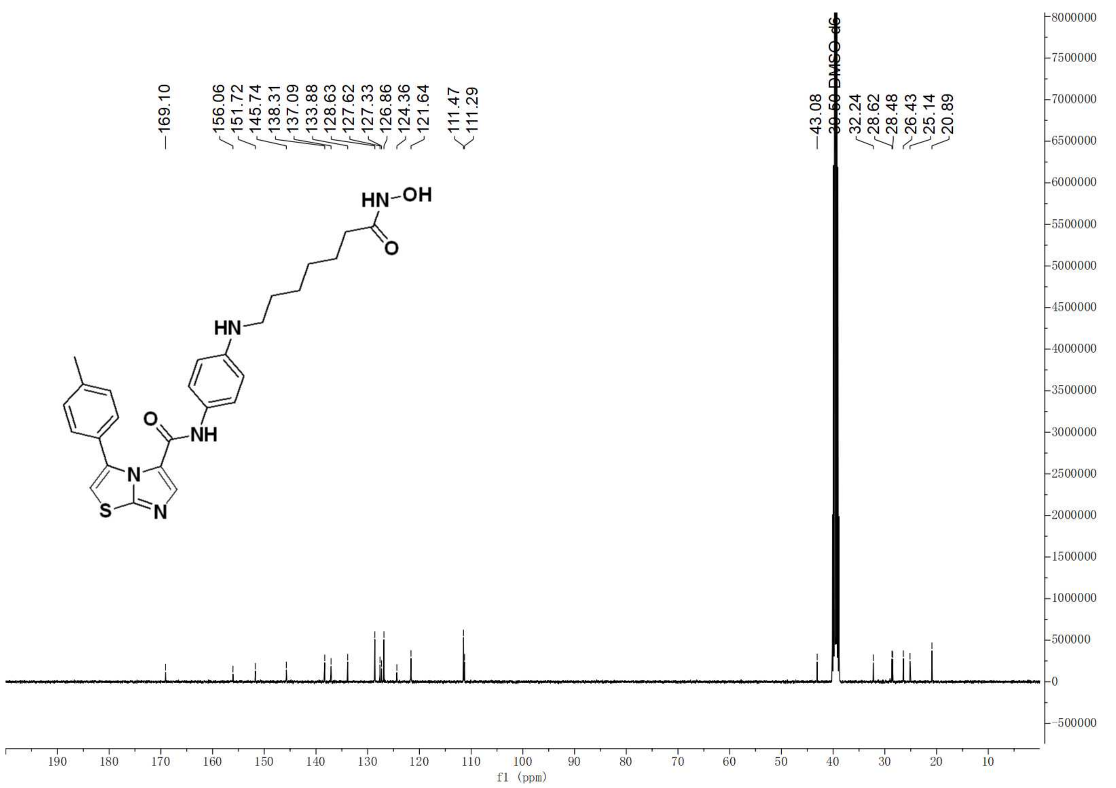

Figure S38. ^{13}C NMR Spectrum of compound **10k**.

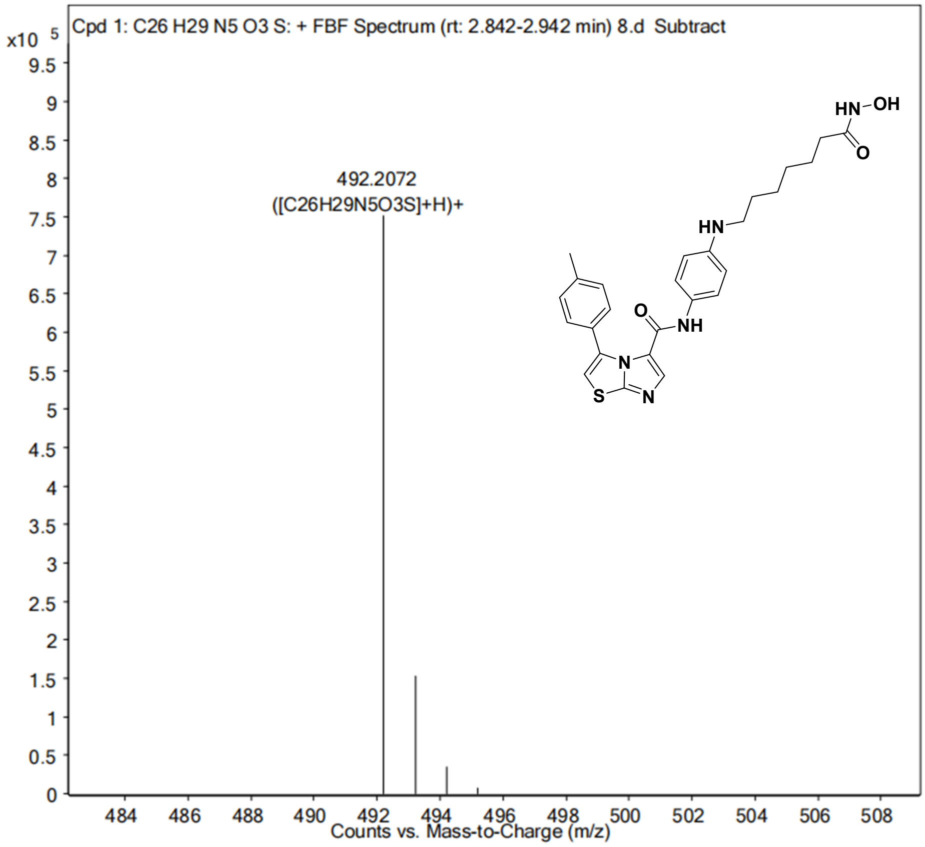

Figure S39. HRMS Spectrum of compound **10k**.

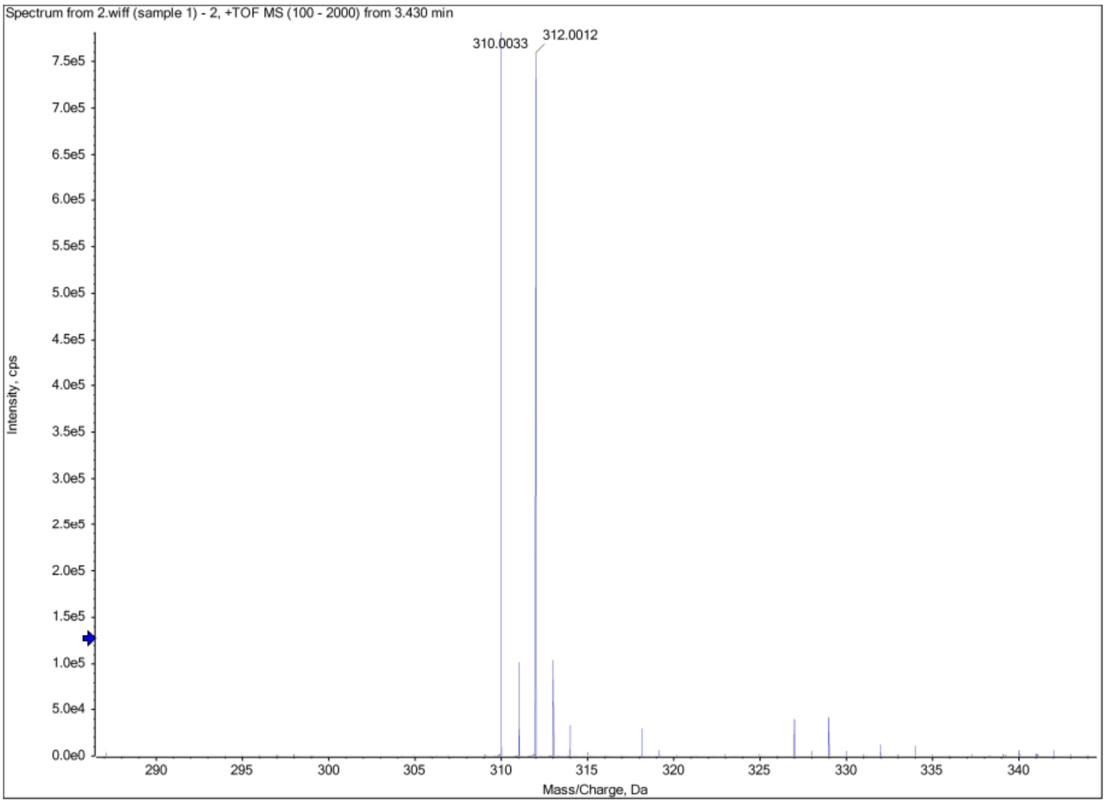

Figure S40. HRMS Spectrum of compound **2a**.

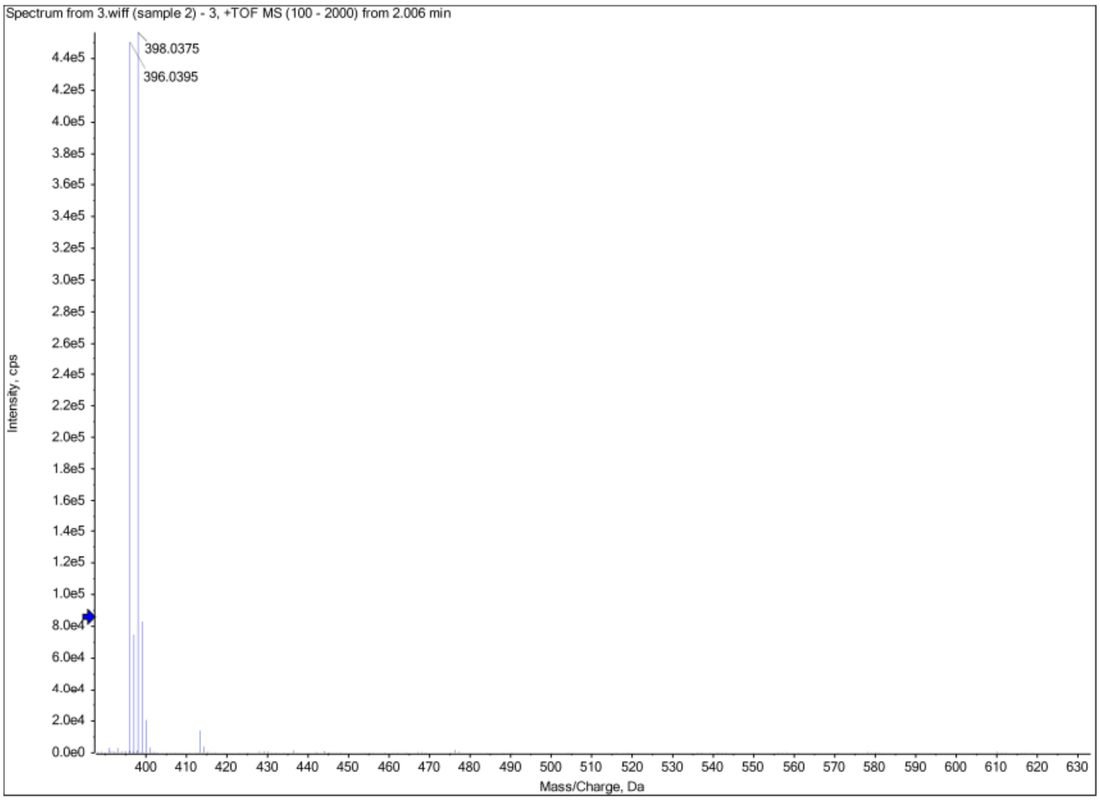

Figure S41. HRMS Spectrum of compound **3a**.

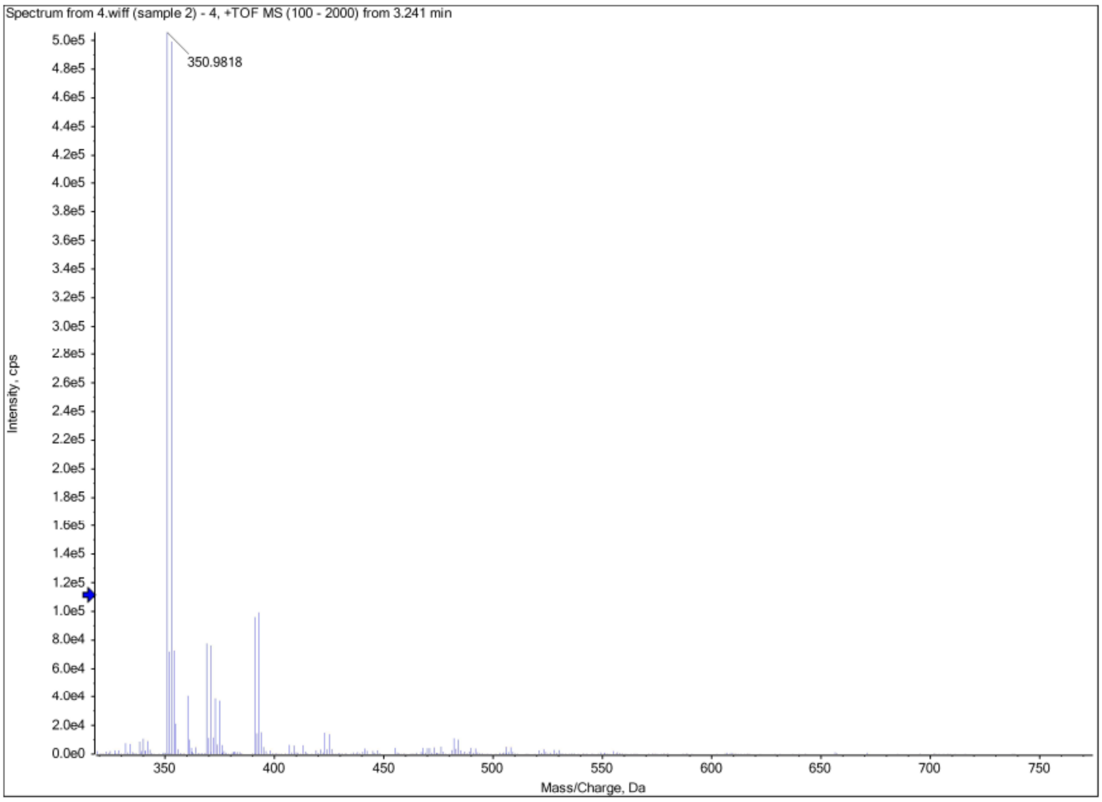

Figure S42. HRMS Spectrum of compound **4a**.

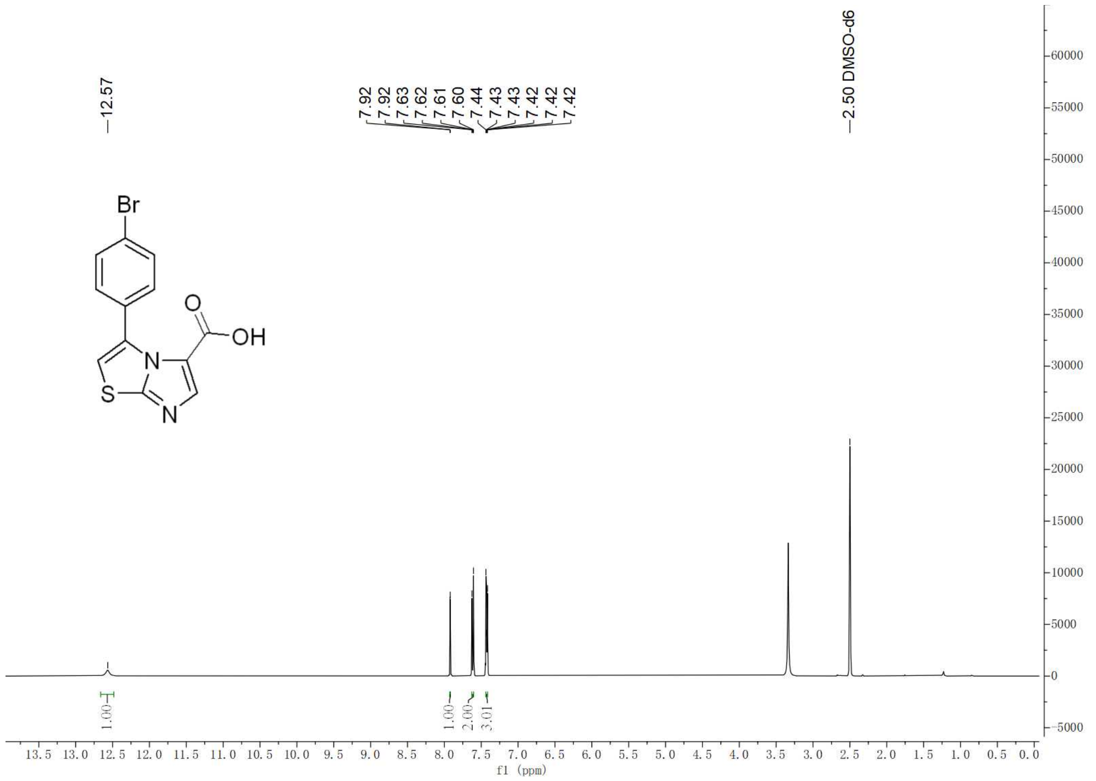

Figure S43. ^{1}H NMR Spectrum of compound **5a**.

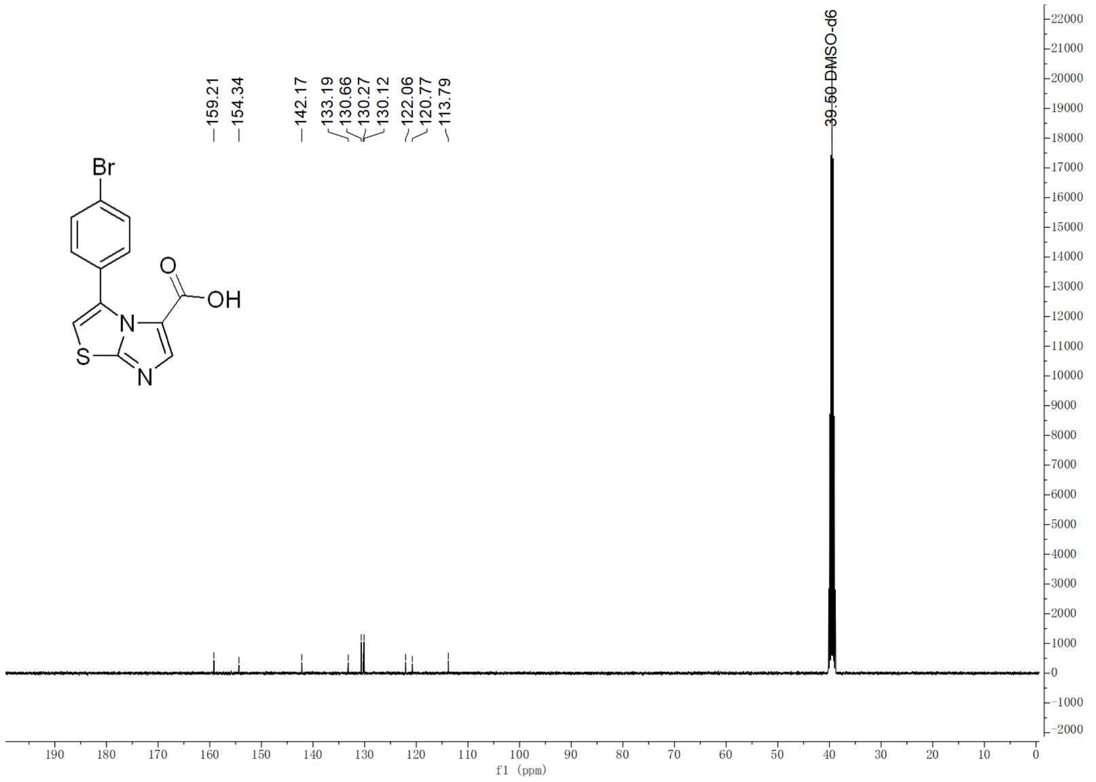

Figure S44. ^{13}C NMR Spectrum of compound **5a**.

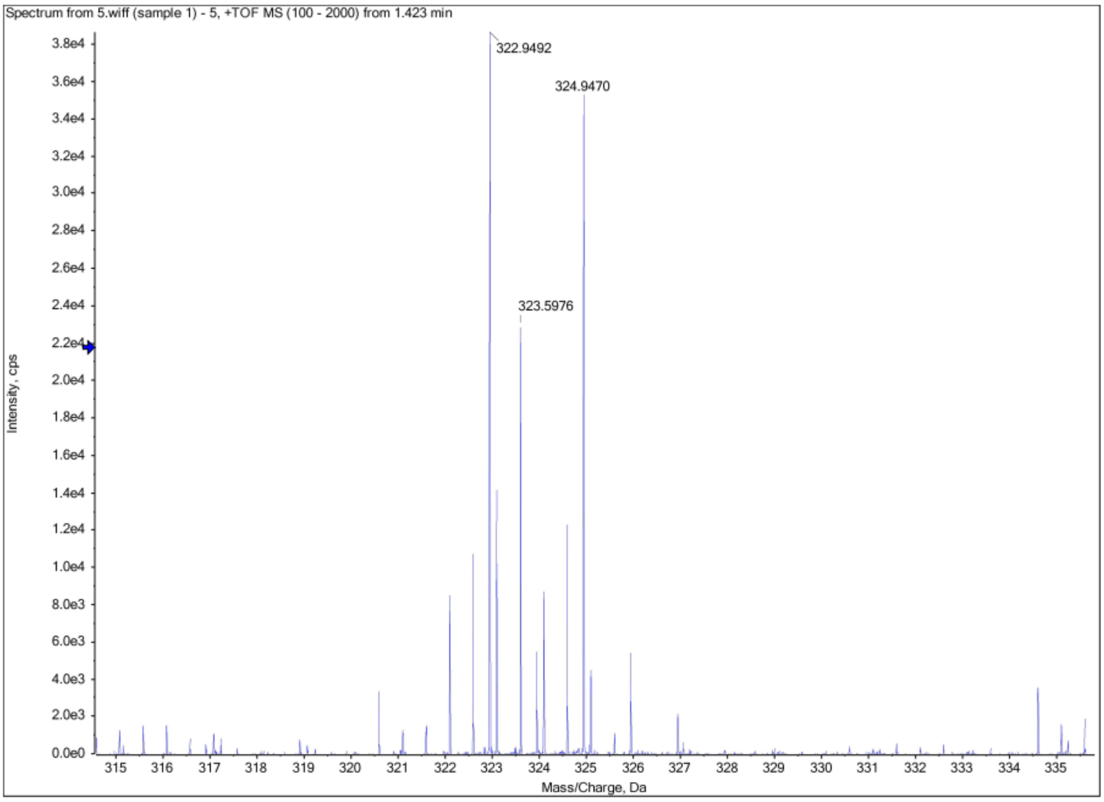

Figure S45. HRMS Spectrum of compound **5a**.

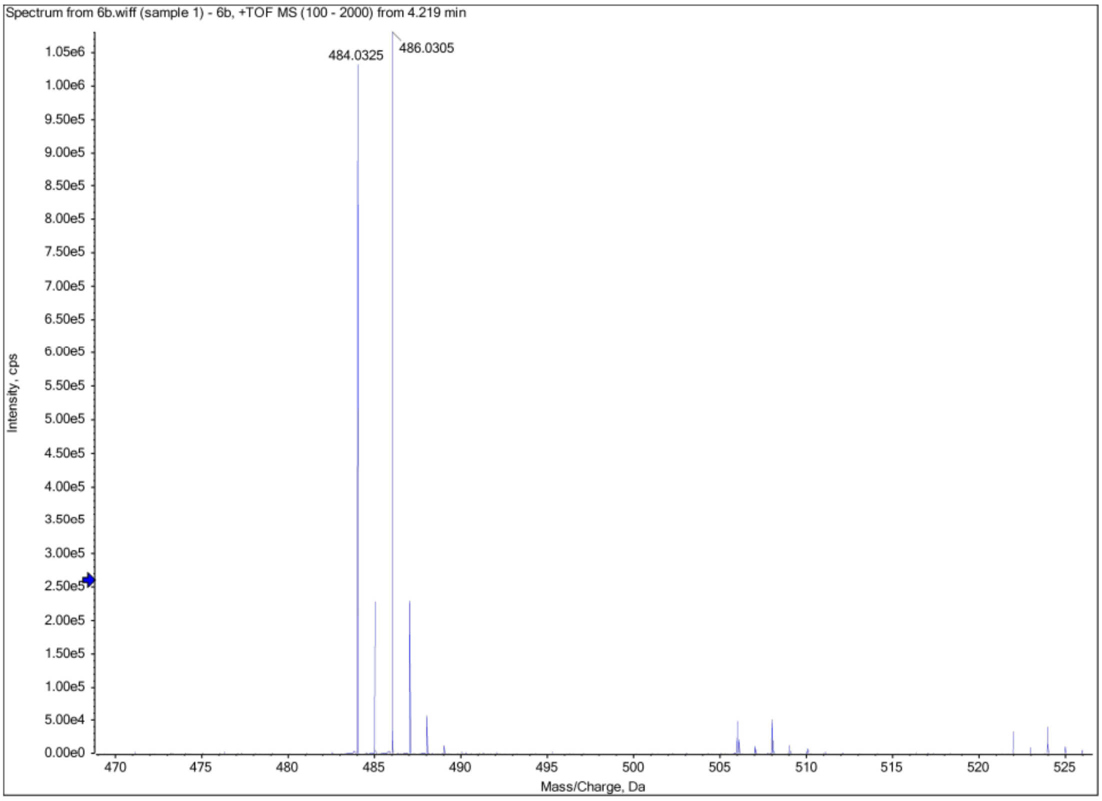

Figure S46. HRMS Spectrum of compound **6b**.

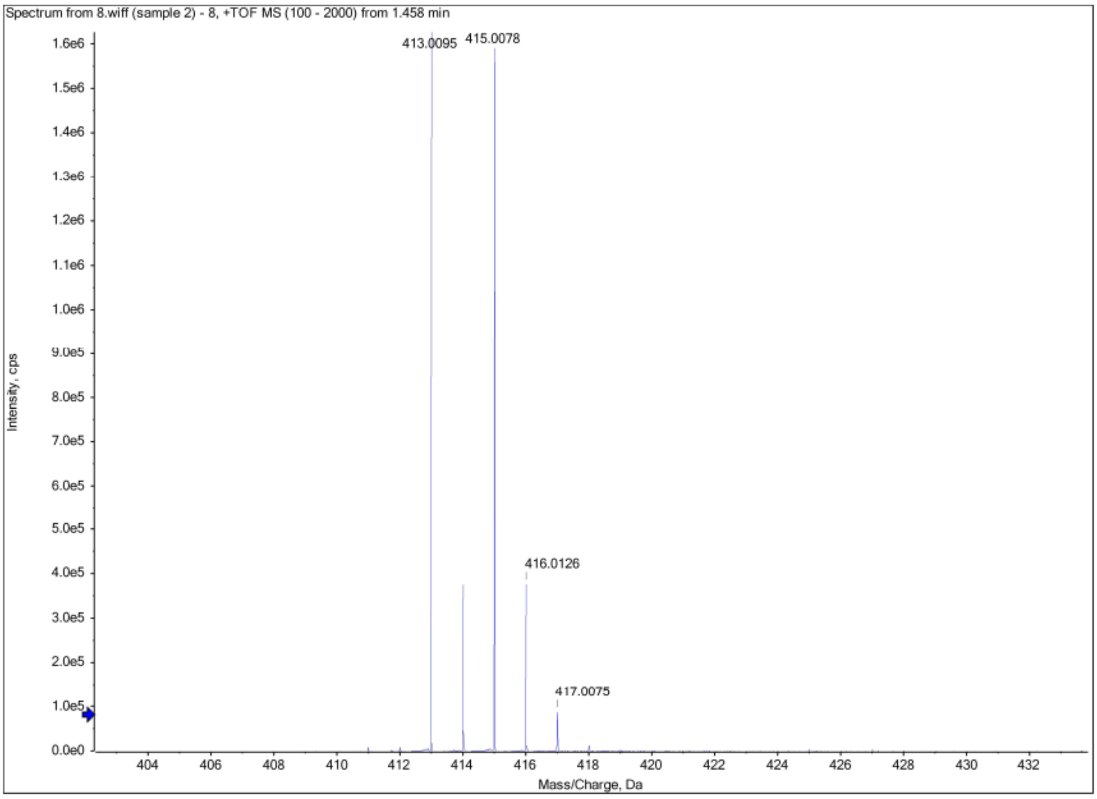

Figure S47. HRMS Spectrum of compound **8**.

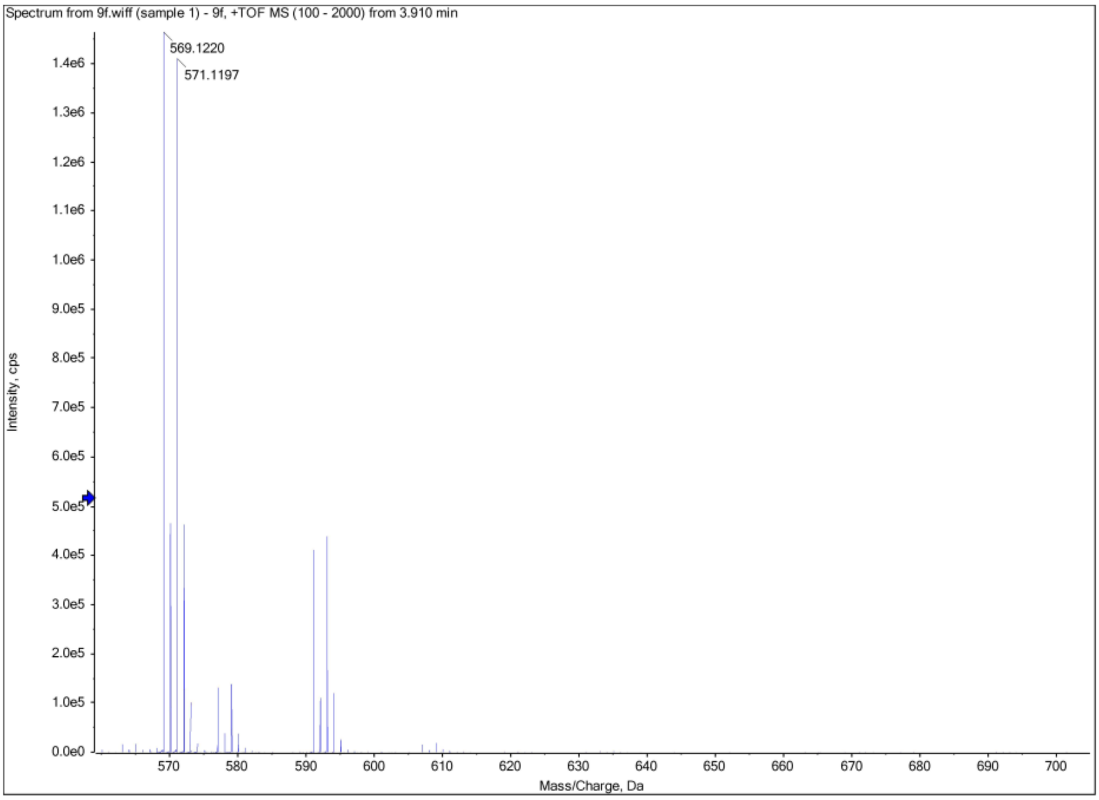

Figure S48. HRMS Spectrum of compound **9f**.

References

15. Tojo, S.; Kohno, T.; Tanaka, T.; Kamioka, S.; Ota, Y.; Ishii, T.; Kamimoto, K.; Asano, S.; Isobe, Y. Crystal Structures and Structure−Activity Relationships of Imidazothiazole Derivatives as IDO1 Inhibitors. *ACS Med. Chem. Lett.* **2014**, *5*, 1119−1123. 15

35. Shen, S.; Svoboda, M.; Zhang, G.; Cavasin, M.A.; Motlova, L.; McKinsey, T.A.; Eubanks, J.H.; Barinka, C.; Kozikowski, A.P. Structural and in Vivo Characterization of Tubastatin A, a Widely Used Histone Deacetylase 6 Inhibitor. *ACS Med. Chem. Lett.* **2020**, *11*, 706-712. 35

42. Huang, R.; Jing, X.; Huang, X.; Pan, Y.; Fang, Y.; Liang, G.; Liao, Z.; Wang, H.; Chen, Z.; Zhang, Y. Bifunctional naphthoquinone aromatic amide-oxime derivatives exert combined immunotherapeutic and antitumor effects through simultaneous targeting of indoleamine-2,3-dioxygenase and signal transducer and activator of transcription 3. *J. Med. Chem.* **2020**, *63*, 1544−1563.

43. Huang, X; Wang, M.; You, Q.; Kong, J.; Zhang, H.; Yu, C.; Wang, Y.; Wang, H.; Huang, R. Synthesis, mechanisms of action, and toxicity of novel aminophosphonates derivatives conjugated irinotecan in vitro and in vivo as potent antitumor agents. *Eur. J. Med. Chem.* **2020**, *189*, 112067.

44. Liang, G.-B.; Wei, J.-H.; Jiang, H.; Huang, R.-Z.; Qin, J.-T.; Wang, H.-L.; Wang, H.-S.; Zhang, Y. Design, synthesis and antitumor evaluation of new 1,8-naphthalimide derivatives targeting nuclear DNA. *Eur. J. Med. Chem.* **2021**, *210*, 112951.
